# Supplementary figures and images for: DANNET: deep attention neural network for efficient ear identification in biometrics (part 2 of 4)
Source: PeerJ Comput Sci. 2024 Dec 18;10:e2603. doi: 10.7717/peerj-cs.2603 (PMC11784740; doi:10.7717/peerj-cs.2603)

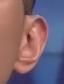

Supplement: Supplemental Information 2 — The EarVN1.0 dataset is a comprehensive collection of over 28,412 ear images from 164 individuals. It encompasses a wide range of variations in pose, scale, illumination, occlusion, resolution, and lighting conditions. This dataset is suitable for various applications such as person authentication and classification. This file contains a sample subset of EarVN1.0 that consists of selected ear images from male participants (sampled from Person IDs 1–98). The images capture a variety of angles, lighting conditions, and backgrounds to ensure diversity and support robust model training for male ear recognition tasks. [file peerj-cs-10-2603-s002.zip › 075.Rym/075 (45).jpg]

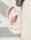

Supplement: Supplemental Information 2 — The EarVN1.0 dataset is a comprehensive collection of over 28,412 ear images from 164 individuals. It encompasses a wide range of variations in pose, scale, illumination, occlusion, resolution, and lighting conditions. This dataset is suitable for various applications such as person authentication and classification. This file contains a sample subset of EarVN1.0 that consists of selected ear images from male participants (sampled from Person IDs 1–98). The images capture a variety of angles, lighting conditions, and backgrounds to ensure diversity and support robust model training for male ear recognition tasks. [file peerj-cs-10-2603-s002.zip › 075.Rym/075 (46).jpg]

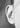

Supplement: Supplemental Information 2 — The EarVN1.0 dataset is a comprehensive collection of over 28,412 ear images from 164 individuals. It encompasses a wide range of variations in pose, scale, illumination, occlusion, resolution, and lighting conditions. This dataset is suitable for various applications such as person authentication and classification. This file contains a sample subset of EarVN1.0 that consists of selected ear images from male participants (sampled from Person IDs 1–98). The images capture a variety of angles, lighting conditions, and backgrounds to ensure diversity and support robust model training for male ear recognition tasks. [file peerj-cs-10-2603-s002.zip › 075.Rym/075 (47).jpg]

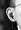

Supplement: Supplemental Information 2 — The EarVN1.0 dataset is a comprehensive collection of over 28,412 ear images from 164 individuals. It encompasses a wide range of variations in pose, scale, illumination, occlusion, resolution, and lighting conditions. This dataset is suitable for various applications such as person authentication and classification. This file contains a sample subset of EarVN1.0 that consists of selected ear images from male participants (sampled from Person IDs 1–98). The images capture a variety of angles, lighting conditions, and backgrounds to ensure diversity and support robust model training for male ear recognition tasks. [file peerj-cs-10-2603-s002.zip › 075.Rym/075 (48).jpg]

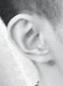

Supplement: Supplemental Information 2 — The EarVN1.0 dataset is a comprehensive collection of over 28,412 ear images from 164 individuals. It encompasses a wide range of variations in pose, scale, illumination, occlusion, resolution, and lighting conditions. This dataset is suitable for various applications such as person authentication and classification. This file contains a sample subset of EarVN1.0 that consists of selected ear images from male participants (sampled from Person IDs 1–98). The images capture a variety of angles, lighting conditions, and backgrounds to ensure diversity and support robust model training for male ear recognition tasks. [file peerj-cs-10-2603-s002.zip › 075.Rym/075 (49).jpg]

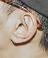

Supplement: Supplemental Information 2 — The EarVN1.0 dataset is a comprehensive collection of over 28,412 ear images from 164 individuals. It encompasses a wide range of variations in pose, scale, illumination, occlusion, resolution, and lighting conditions. This dataset is suitable for various applications such as person authentication and classification. This file contains a sample subset of EarVN1.0 that consists of selected ear images from male participants (sampled from Person IDs 1–98). The images capture a variety of angles, lighting conditions, and backgrounds to ensure diversity and support robust model training for male ear recognition tasks. [file peerj-cs-10-2603-s002.zip › 075.Rym/075 (5).jpg]

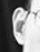

Supplement: Supplemental Information 2 — The EarVN1.0 dataset is a comprehensive collection of over 28,412 ear images from 164 individuals. It encompasses a wide range of variations in pose, scale, illumination, occlusion, resolution, and lighting conditions. This dataset is suitable for various applications such as person authentication and classification. This file contains a sample subset of EarVN1.0 that consists of selected ear images from male participants (sampled from Person IDs 1–98). The images capture a variety of angles, lighting conditions, and backgrounds to ensure diversity and support robust model training for male ear recognition tasks. [file peerj-cs-10-2603-s002.zip › 075.Rym/075 (50).jpg]

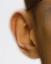

Supplement: Supplemental Information 2 — The EarVN1.0 dataset is a comprehensive collection of over 28,412 ear images from 164 individuals. It encompasses a wide range of variations in pose, scale, illumination, occlusion, resolution, and lighting conditions. This dataset is suitable for various applications such as person authentication and classification. This file contains a sample subset of EarVN1.0 that consists of selected ear images from male participants (sampled from Person IDs 1–98). The images capture a variety of angles, lighting conditions, and backgrounds to ensure diversity and support robust model training for male ear recognition tasks. [file peerj-cs-10-2603-s002.zip › 075.Rym/075 (51).jpg]

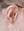

Supplement: Supplemental Information 2 — The EarVN1.0 dataset is a comprehensive collection of over 28,412 ear images from 164 individuals. It encompasses a wide range of variations in pose, scale, illumination, occlusion, resolution, and lighting conditions. This dataset is suitable for various applications such as person authentication and classification. This file contains a sample subset of EarVN1.0 that consists of selected ear images from male participants (sampled from Person IDs 1–98). The images capture a variety of angles, lighting conditions, and backgrounds to ensure diversity and support robust model training for male ear recognition tasks. [file peerj-cs-10-2603-s002.zip › 075.Rym/075 (52).jpg]

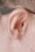

Supplement: Supplemental Information 2 — The EarVN1.0 dataset is a comprehensive collection of over 28,412 ear images from 164 individuals. It encompasses a wide range of variations in pose, scale, illumination, occlusion, resolution, and lighting conditions. This dataset is suitable for various applications such as person authentication and classification. This file contains a sample subset of EarVN1.0 that consists of selected ear images from male participants (sampled from Person IDs 1–98). The images capture a variety of angles, lighting conditions, and backgrounds to ensure diversity and support robust model training for male ear recognition tasks. [file peerj-cs-10-2603-s002.zip › 075.Rym/075 (53).jpg]

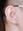

Supplement: Supplemental Information 2 — The EarVN1.0 dataset is a comprehensive collection of over 28,412 ear images from 164 individuals. It encompasses a wide range of variations in pose, scale, illumination, occlusion, resolution, and lighting conditions. This dataset is suitable for various applications such as person authentication and classification. This file contains a sample subset of EarVN1.0 that consists of selected ear images from male participants (sampled from Person IDs 1–98). The images capture a variety of angles, lighting conditions, and backgrounds to ensure diversity and support robust model training for male ear recognition tasks. [file peerj-cs-10-2603-s002.zip › 075.Rym/075 (54).jpg]

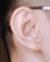

Supplement: Supplemental Information 2 — The EarVN1.0 dataset is a comprehensive collection of over 28,412 ear images from 164 individuals. It encompasses a wide range of variations in pose, scale, illumination, occlusion, resolution, and lighting conditions. This dataset is suitable for various applications such as person authentication and classification. This file contains a sample subset of EarVN1.0 that consists of selected ear images from male participants (sampled from Person IDs 1–98). The images capture a variety of angles, lighting conditions, and backgrounds to ensure diversity and support robust model training for male ear recognition tasks. [file peerj-cs-10-2603-s002.zip › 075.Rym/075 (55).jpg]

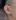

Supplement: Supplemental Information 2 — The EarVN1.0 dataset is a comprehensive collection of over 28,412 ear images from 164 individuals. It encompasses a wide range of variations in pose, scale, illumination, occlusion, resolution, and lighting conditions. This dataset is suitable for various applications such as person authentication and classification. This file contains a sample subset of EarVN1.0 that consists of selected ear images from male participants (sampled from Person IDs 1–98). The images capture a variety of angles, lighting conditions, and backgrounds to ensure diversity and support robust model training for male ear recognition tasks. [file peerj-cs-10-2603-s002.zip › 075.Rym/075 (56).jpg]

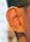

Supplement: Supplemental Information 2 — The EarVN1.0 dataset is a comprehensive collection of over 28,412 ear images from 164 individuals. It encompasses a wide range of variations in pose, scale, illumination, occlusion, resolution, and lighting conditions. This dataset is suitable for various applications such as person authentication and classification. This file contains a sample subset of EarVN1.0 that consists of selected ear images from male participants (sampled from Person IDs 1–98). The images capture a variety of angles, lighting conditions, and backgrounds to ensure diversity and support robust model training for male ear recognition tasks. [file peerj-cs-10-2603-s002.zip › 075.Rym/075 (57).jpg]

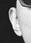

Supplement: Supplemental Information 2 — The EarVN1.0 dataset is a comprehensive collection of over 28,412 ear images from 164 individuals. It encompasses a wide range of variations in pose, scale, illumination, occlusion, resolution, and lighting conditions. This dataset is suitable for various applications such as person authentication and classification. This file contains a sample subset of EarVN1.0 that consists of selected ear images from male participants (sampled from Person IDs 1–98). The images capture a variety of angles, lighting conditions, and backgrounds to ensure diversity and support robust model training for male ear recognition tasks. [file peerj-cs-10-2603-s002.zip › 075.Rym/075 (58).jpg]

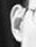

Supplement: Supplemental Information 2 — The EarVN1.0 dataset is a comprehensive collection of over 28,412 ear images from 164 individuals. It encompasses a wide range of variations in pose, scale, illumination, occlusion, resolution, and lighting conditions. This dataset is suitable for various applications such as person authentication and classification. This file contains a sample subset of EarVN1.0 that consists of selected ear images from male participants (sampled from Person IDs 1–98). The images capture a variety of angles, lighting conditions, and backgrounds to ensure diversity and support robust model training for male ear recognition tasks. [file peerj-cs-10-2603-s002.zip › 075.Rym/075 (59).jpg]

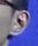

Supplement: Supplemental Information 2 — The EarVN1.0 dataset is a comprehensive collection of over 28,412 ear images from 164 individuals. It encompasses a wide range of variations in pose, scale, illumination, occlusion, resolution, and lighting conditions. This dataset is suitable for various applications such as person authentication and classification. This file contains a sample subset of EarVN1.0 that consists of selected ear images from male participants (sampled from Person IDs 1–98). The images capture a variety of angles, lighting conditions, and backgrounds to ensure diversity and support robust model training for male ear recognition tasks. [file peerj-cs-10-2603-s002.zip › 075.Rym/075 (6).jpg]

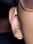

Supplement: Supplemental Information 2 — The EarVN1.0 dataset is a comprehensive collection of over 28,412 ear images from 164 individuals. It encompasses a wide range of variations in pose, scale, illumination, occlusion, resolution, and lighting conditions. This dataset is suitable for various applications such as person authentication and classification. This file contains a sample subset of EarVN1.0 that consists of selected ear images from male participants (sampled from Person IDs 1–98). The images capture a variety of angles, lighting conditions, and backgrounds to ensure diversity and support robust model training for male ear recognition tasks. [file peerj-cs-10-2603-s002.zip › 075.Rym/075 (60).jpg]

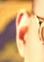

Supplement: Supplemental Information 2 — The EarVN1.0 dataset is a comprehensive collection of over 28,412 ear images from 164 individuals. It encompasses a wide range of variations in pose, scale, illumination, occlusion, resolution, and lighting conditions. This dataset is suitable for various applications such as person authentication and classification. This file contains a sample subset of EarVN1.0 that consists of selected ear images from male participants (sampled from Person IDs 1–98). The images capture a variety of angles, lighting conditions, and backgrounds to ensure diversity and support robust model training for male ear recognition tasks. [file peerj-cs-10-2603-s002.zip › 075.Rym/075 (61).jpg]

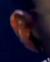

Supplement: Supplemental Information 2 — The EarVN1.0 dataset is a comprehensive collection of over 28,412 ear images from 164 individuals. It encompasses a wide range of variations in pose, scale, illumination, occlusion, resolution, and lighting conditions. This dataset is suitable for various applications such as person authentication and classification. This file contains a sample subset of EarVN1.0 that consists of selected ear images from male participants (sampled from Person IDs 1–98). The images capture a variety of angles, lighting conditions, and backgrounds to ensure diversity and support robust model training for male ear recognition tasks. [file peerj-cs-10-2603-s002.zip › 075.Rym/075 (62).jpg]

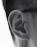

Supplement: Supplemental Information 2 — The EarVN1.0 dataset is a comprehensive collection of over 28,412 ear images from 164 individuals. It encompasses a wide range of variations in pose, scale, illumination, occlusion, resolution, and lighting conditions. This dataset is suitable for various applications such as person authentication and classification. This file contains a sample subset of EarVN1.0 that consists of selected ear images from male participants (sampled from Person IDs 1–98). The images capture a variety of angles, lighting conditions, and backgrounds to ensure diversity and support robust model training for male ear recognition tasks. [file peerj-cs-10-2603-s002.zip › 075.Rym/075 (63).jpg]

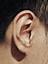

Supplement: Supplemental Information 2 — The EarVN1.0 dataset is a comprehensive collection of over 28,412 ear images from 164 individuals. It encompasses a wide range of variations in pose, scale, illumination, occlusion, resolution, and lighting conditions. This dataset is suitable for various applications such as person authentication and classification. This file contains a sample subset of EarVN1.0 that consists of selected ear images from male participants (sampled from Person IDs 1–98). The images capture a variety of angles, lighting conditions, and backgrounds to ensure diversity and support robust model training for male ear recognition tasks. [file peerj-cs-10-2603-s002.zip › 075.Rym/075 (64).jpg]

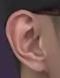

Supplement: Supplemental Information 2 — The EarVN1.0 dataset is a comprehensive collection of over 28,412 ear images from 164 individuals. It encompasses a wide range of variations in pose, scale, illumination, occlusion, resolution, and lighting conditions. This dataset is suitable for various applications such as person authentication and classification. This file contains a sample subset of EarVN1.0 that consists of selected ear images from male participants (sampled from Person IDs 1–98). The images capture a variety of angles, lighting conditions, and backgrounds to ensure diversity and support robust model training for male ear recognition tasks. [file peerj-cs-10-2603-s002.zip › 075.Rym/075 (65).jpg]

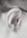

Supplement: Supplemental Information 2 — The EarVN1.0 dataset is a comprehensive collection of over 28,412 ear images from 164 individuals. It encompasses a wide range of variations in pose, scale, illumination, occlusion, resolution, and lighting conditions. This dataset is suitable for various applications such as person authentication and classification. This file contains a sample subset of EarVN1.0 that consists of selected ear images from male participants (sampled from Person IDs 1–98). The images capture a variety of angles, lighting conditions, and backgrounds to ensure diversity and support robust model training for male ear recognition tasks. [file peerj-cs-10-2603-s002.zip › 075.Rym/075 (66).jpg]

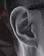

Supplement: Supplemental Information 2 — The EarVN1.0 dataset is a comprehensive collection of over 28,412 ear images from 164 individuals. It encompasses a wide range of variations in pose, scale, illumination, occlusion, resolution, and lighting conditions. This dataset is suitable for various applications such as person authentication and classification. This file contains a sample subset of EarVN1.0 that consists of selected ear images from male participants (sampled from Person IDs 1–98). The images capture a variety of angles, lighting conditions, and backgrounds to ensure diversity and support robust model training for male ear recognition tasks. [file peerj-cs-10-2603-s002.zip › 075.Rym/075 (67).jpg]

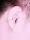

Supplement: Supplemental Information 2 — The EarVN1.0 dataset is a comprehensive collection of over 28,412 ear images from 164 individuals. It encompasses a wide range of variations in pose, scale, illumination, occlusion, resolution, and lighting conditions. This dataset is suitable for various applications such as person authentication and classification. This file contains a sample subset of EarVN1.0 that consists of selected ear images from male participants (sampled from Person IDs 1–98). The images capture a variety of angles, lighting conditions, and backgrounds to ensure diversity and support robust model training for male ear recognition tasks. [file peerj-cs-10-2603-s002.zip › 075.Rym/075 (68).jpg]

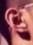

Supplement: Supplemental Information 2 — The EarVN1.0 dataset is a comprehensive collection of over 28,412 ear images from 164 individuals. It encompasses a wide range of variations in pose, scale, illumination, occlusion, resolution, and lighting conditions. This dataset is suitable for various applications such as person authentication and classification. This file contains a sample subset of EarVN1.0 that consists of selected ear images from male participants (sampled from Person IDs 1–98). The images capture a variety of angles, lighting conditions, and backgrounds to ensure diversity and support robust model training for male ear recognition tasks. [file peerj-cs-10-2603-s002.zip › 075.Rym/075 (69).jpg]

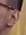

Supplement: Supplemental Information 2 — The EarVN1.0 dataset is a comprehensive collection of over 28,412 ear images from 164 individuals. It encompasses a wide range of variations in pose, scale, illumination, occlusion, resolution, and lighting conditions. This dataset is suitable for various applications such as person authentication and classification. This file contains a sample subset of EarVN1.0 that consists of selected ear images from male participants (sampled from Person IDs 1–98). The images capture a variety of angles, lighting conditions, and backgrounds to ensure diversity and support robust model training for male ear recognition tasks. [file peerj-cs-10-2603-s002.zip › 075.Rym/075 (7).jpg]

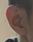

Supplement: Supplemental Information 2 — The EarVN1.0 dataset is a comprehensive collection of over 28,412 ear images from 164 individuals. It encompasses a wide range of variations in pose, scale, illumination, occlusion, resolution, and lighting conditions. This dataset is suitable for various applications such as person authentication and classification. This file contains a sample subset of EarVN1.0 that consists of selected ear images from male participants (sampled from Person IDs 1–98). The images capture a variety of angles, lighting conditions, and backgrounds to ensure diversity and support robust model training for male ear recognition tasks. [file peerj-cs-10-2603-s002.zip › 075.Rym/075 (70).jpg]

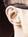

Supplement: Supplemental Information 2 — The EarVN1.0 dataset is a comprehensive collection of over 28,412 ear images from 164 individuals. It encompasses a wide range of variations in pose, scale, illumination, occlusion, resolution, and lighting conditions. This dataset is suitable for various applications such as person authentication and classification. This file contains a sample subset of EarVN1.0 that consists of selected ear images from male participants (sampled from Person IDs 1–98). The images capture a variety of angles, lighting conditions, and backgrounds to ensure diversity and support robust model training for male ear recognition tasks. [file peerj-cs-10-2603-s002.zip › 075.Rym/075 (71).jpg]

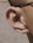

Supplement: Supplemental Information 2 — The EarVN1.0 dataset is a comprehensive collection of over 28,412 ear images from 164 individuals. It encompasses a wide range of variations in pose, scale, illumination, occlusion, resolution, and lighting conditions. This dataset is suitable for various applications such as person authentication and classification. This file contains a sample subset of EarVN1.0 that consists of selected ear images from male participants (sampled from Person IDs 1–98). The images capture a variety of angles, lighting conditions, and backgrounds to ensure diversity and support robust model training for male ear recognition tasks. [file peerj-cs-10-2603-s002.zip › 075.Rym/075 (72).jpg]

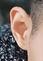

Supplement: Supplemental Information 2 — The EarVN1.0 dataset is a comprehensive collection of over 28,412 ear images from 164 individuals. It encompasses a wide range of variations in pose, scale, illumination, occlusion, resolution, and lighting conditions. This dataset is suitable for various applications such as person authentication and classification. This file contains a sample subset of EarVN1.0 that consists of selected ear images from male participants (sampled from Person IDs 1–98). The images capture a variety of angles, lighting conditions, and backgrounds to ensure diversity and support robust model training for male ear recognition tasks. [file peerj-cs-10-2603-s002.zip › 075.Rym/075 (73).jpg]

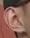

Supplement: Supplemental Information 2 — The EarVN1.0 dataset is a comprehensive collection of over 28,412 ear images from 164 individuals. It encompasses a wide range of variations in pose, scale, illumination, occlusion, resolution, and lighting conditions. This dataset is suitable for various applications such as person authentication and classification. This file contains a sample subset of EarVN1.0 that consists of selected ear images from male participants (sampled from Person IDs 1–98). The images capture a variety of angles, lighting conditions, and backgrounds to ensure diversity and support robust model training for male ear recognition tasks. [file peerj-cs-10-2603-s002.zip › 075.Rym/075 (74).jpg]

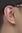

Supplement: Supplemental Information 2 — The EarVN1.0 dataset is a comprehensive collection of over 28,412 ear images from 164 individuals. It encompasses a wide range of variations in pose, scale, illumination, occlusion, resolution, and lighting conditions. This dataset is suitable for various applications such as person authentication and classification. This file contains a sample subset of EarVN1.0 that consists of selected ear images from male participants (sampled from Person IDs 1–98). The images capture a variety of angles, lighting conditions, and backgrounds to ensure diversity and support robust model training for male ear recognition tasks. [file peerj-cs-10-2603-s002.zip › 075.Rym/075 (75).jpg]

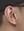

Supplement: Supplemental Information 2 — The EarVN1.0 dataset is a comprehensive collection of over 28,412 ear images from 164 individuals. It encompasses a wide range of variations in pose, scale, illumination, occlusion, resolution, and lighting conditions. This dataset is suitable for various applications such as person authentication and classification. This file contains a sample subset of EarVN1.0 that consists of selected ear images from male participants (sampled from Person IDs 1–98). The images capture a variety of angles, lighting conditions, and backgrounds to ensure diversity and support robust model training for male ear recognition tasks. [file peerj-cs-10-2603-s002.zip › 075.Rym/075 (76).jpg]

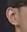

Supplement: Supplemental Information 2 — The EarVN1.0 dataset is a comprehensive collection of over 28,412 ear images from 164 individuals. It encompasses a wide range of variations in pose, scale, illumination, occlusion, resolution, and lighting conditions. This dataset is suitable for various applications such as person authentication and classification. This file contains a sample subset of EarVN1.0 that consists of selected ear images from male participants (sampled from Person IDs 1–98). The images capture a variety of angles, lighting conditions, and backgrounds to ensure diversity and support robust model training for male ear recognition tasks. [file peerj-cs-10-2603-s002.zip › 075.Rym/075 (77).jpg]

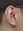

Supplement: Supplemental Information 2 — The EarVN1.0 dataset is a comprehensive collection of over 28,412 ear images from 164 individuals. It encompasses a wide range of variations in pose, scale, illumination, occlusion, resolution, and lighting conditions. This dataset is suitable for various applications such as person authentication and classification. This file contains a sample subset of EarVN1.0 that consists of selected ear images from male participants (sampled from Person IDs 1–98). The images capture a variety of angles, lighting conditions, and backgrounds to ensure diversity and support robust model training for male ear recognition tasks. [file peerj-cs-10-2603-s002.zip › 075.Rym/075 (78).jpg]

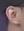

Supplement: Supplemental Information 2 — The EarVN1.0 dataset is a comprehensive collection of over 28,412 ear images from 164 individuals. It encompasses a wide range of variations in pose, scale, illumination, occlusion, resolution, and lighting conditions. This dataset is suitable for various applications such as person authentication and classification. This file contains a sample subset of EarVN1.0 that consists of selected ear images from male participants (sampled from Person IDs 1–98). The images capture a variety of angles, lighting conditions, and backgrounds to ensure diversity and support robust model training for male ear recognition tasks. [file peerj-cs-10-2603-s002.zip › 075.Rym/075 (79).jpg]

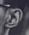

Supplement: Supplemental Information 2 — The EarVN1.0 dataset is a comprehensive collection of over 28,412 ear images from 164 individuals. It encompasses a wide range of variations in pose, scale, illumination, occlusion, resolution, and lighting conditions. This dataset is suitable for various applications such as person authentication and classification. This file contains a sample subset of EarVN1.0 that consists of selected ear images from male participants (sampled from Person IDs 1–98). The images capture a variety of angles, lighting conditions, and backgrounds to ensure diversity and support robust model training for male ear recognition tasks. [file peerj-cs-10-2603-s002.zip › 075.Rym/075 (8).jpg]

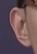

Supplement: Supplemental Information 2 — The EarVN1.0 dataset is a comprehensive collection of over 28,412 ear images from 164 individuals. It encompasses a wide range of variations in pose, scale, illumination, occlusion, resolution, and lighting conditions. This dataset is suitable for various applications such as person authentication and classification. This file contains a sample subset of EarVN1.0 that consists of selected ear images from male participants (sampled from Person IDs 1–98). The images capture a variety of angles, lighting conditions, and backgrounds to ensure diversity and support robust model training for male ear recognition tasks. [file peerj-cs-10-2603-s002.zip › 075.Rym/075 (80).jpg]

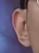

Supplement: Supplemental Information 2 — The EarVN1.0 dataset is a comprehensive collection of over 28,412 ear images from 164 individuals. It encompasses a wide range of variations in pose, scale, illumination, occlusion, resolution, and lighting conditions. This dataset is suitable for various applications such as person authentication and classification. This file contains a sample subset of EarVN1.0 that consists of selected ear images from male participants (sampled from Person IDs 1–98). The images capture a variety of angles, lighting conditions, and backgrounds to ensure diversity and support robust model training for male ear recognition tasks. [file peerj-cs-10-2603-s002.zip › 075.Rym/075 (81).jpg]

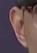

Supplement: Supplemental Information 2 — The EarVN1.0 dataset is a comprehensive collection of over 28,412 ear images from 164 individuals. It encompasses a wide range of variations in pose, scale, illumination, occlusion, resolution, and lighting conditions. This dataset is suitable for various applications such as person authentication and classification. This file contains a sample subset of EarVN1.0 that consists of selected ear images from male participants (sampled from Person IDs 1–98). The images capture a variety of angles, lighting conditions, and backgrounds to ensure diversity and support robust model training for male ear recognition tasks. [file peerj-cs-10-2603-s002.zip › 075.Rym/075 (82).jpg]

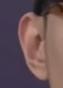

Supplement: Supplemental Information 2 — The EarVN1.0 dataset is a comprehensive collection of over 28,412 ear images from 164 individuals. It encompasses a wide range of variations in pose, scale, illumination, occlusion, resolution, and lighting conditions. This dataset is suitable for various applications such as person authentication and classification. This file contains a sample subset of EarVN1.0 that consists of selected ear images from male participants (sampled from Person IDs 1–98). The images capture a variety of angles, lighting conditions, and backgrounds to ensure diversity and support robust model training for male ear recognition tasks. [file peerj-cs-10-2603-s002.zip › 075.Rym/075 (83).jpg]

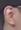

Supplement: Supplemental Information 2 — The EarVN1.0 dataset is a comprehensive collection of over 28,412 ear images from 164 individuals. It encompasses a wide range of variations in pose, scale, illumination, occlusion, resolution, and lighting conditions. This dataset is suitable for various applications such as person authentication and classification. This file contains a sample subset of EarVN1.0 that consists of selected ear images from male participants (sampled from Person IDs 1–98). The images capture a variety of angles, lighting conditions, and backgrounds to ensure diversity and support robust model training for male ear recognition tasks. [file peerj-cs-10-2603-s002.zip › 075.Rym/075 (84).jpg]

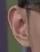

Supplement: Supplemental Information 2 — The EarVN1.0 dataset is a comprehensive collection of over 28,412 ear images from 164 individuals. It encompasses a wide range of variations in pose, scale, illumination, occlusion, resolution, and lighting conditions. This dataset is suitable for various applications such as person authentication and classification. This file contains a sample subset of EarVN1.0 that consists of selected ear images from male participants (sampled from Person IDs 1–98). The images capture a variety of angles, lighting conditions, and backgrounds to ensure diversity and support robust model training for male ear recognition tasks. [file peerj-cs-10-2603-s002.zip › 075.Rym/075 (85).jpg]

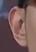

Supplement: Supplemental Information 2 — The EarVN1.0 dataset is a comprehensive collection of over 28,412 ear images from 164 individuals. It encompasses a wide range of variations in pose, scale, illumination, occlusion, resolution, and lighting conditions. This dataset is suitable for various applications such as person authentication and classification. This file contains a sample subset of EarVN1.0 that consists of selected ear images from male participants (sampled from Person IDs 1–98). The images capture a variety of angles, lighting conditions, and backgrounds to ensure diversity and support robust model training for male ear recognition tasks. [file peerj-cs-10-2603-s002.zip › 075.Rym/075 (86).jpg]

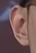

Supplement: Supplemental Information 2 — The EarVN1.0 dataset is a comprehensive collection of over 28,412 ear images from 164 individuals. It encompasses a wide range of variations in pose, scale, illumination, occlusion, resolution, and lighting conditions. This dataset is suitable for various applications such as person authentication and classification. This file contains a sample subset of EarVN1.0 that consists of selected ear images from male participants (sampled from Person IDs 1–98). The images capture a variety of angles, lighting conditions, and backgrounds to ensure diversity and support robust model training for male ear recognition tasks. [file peerj-cs-10-2603-s002.zip › 075.Rym/075 (87).jpg]

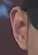

Supplement: Supplemental Information 2 — The EarVN1.0 dataset is a comprehensive collection of over 28,412 ear images from 164 individuals. It encompasses a wide range of variations in pose, scale, illumination, occlusion, resolution, and lighting conditions. This dataset is suitable for various applications such as person authentication and classification. This file contains a sample subset of EarVN1.0 that consists of selected ear images from male participants (sampled from Person IDs 1–98). The images capture a variety of angles, lighting conditions, and backgrounds to ensure diversity and support robust model training for male ear recognition tasks. [file peerj-cs-10-2603-s002.zip › 075.Rym/075 (88).jpg]

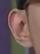

Supplement: Supplemental Information 2 — The EarVN1.0 dataset is a comprehensive collection of over 28,412 ear images from 164 individuals. It encompasses a wide range of variations in pose, scale, illumination, occlusion, resolution, and lighting conditions. This dataset is suitable for various applications such as person authentication and classification. This file contains a sample subset of EarVN1.0 that consists of selected ear images from male participants (sampled from Person IDs 1–98). The images capture a variety of angles, lighting conditions, and backgrounds to ensure diversity and support robust model training for male ear recognition tasks. [file peerj-cs-10-2603-s002.zip › 075.Rym/075 (89).jpg]

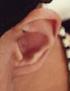

Supplement: Supplemental Information 2 — The EarVN1.0 dataset is a comprehensive collection of over 28,412 ear images from 164 individuals. It encompasses a wide range of variations in pose, scale, illumination, occlusion, resolution, and lighting conditions. This dataset is suitable for various applications such as person authentication and classification. This file contains a sample subset of EarVN1.0 that consists of selected ear images from male participants (sampled from Person IDs 1–98). The images capture a variety of angles, lighting conditions, and backgrounds to ensure diversity and support robust model training for male ear recognition tasks. [file peerj-cs-10-2603-s002.zip › 075.Rym/075 (9).jpg]

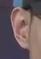

Supplement: Supplemental Information 2 — The EarVN1.0 dataset is a comprehensive collection of over 28,412 ear images from 164 individuals. It encompasses a wide range of variations in pose, scale, illumination, occlusion, resolution, and lighting conditions. This dataset is suitable for various applications such as person authentication and classification. This file contains a sample subset of EarVN1.0 that consists of selected ear images from male participants (sampled from Person IDs 1–98). The images capture a variety of angles, lighting conditions, and backgrounds to ensure diversity and support robust model training for male ear recognition tasks. [file peerj-cs-10-2603-s002.zip › 075.Rym/075 (90).jpg]

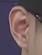

Supplement: Supplemental Information 2 — The EarVN1.0 dataset is a comprehensive collection of over 28,412 ear images from 164 individuals. It encompasses a wide range of variations in pose, scale, illumination, occlusion, resolution, and lighting conditions. This dataset is suitable for various applications such as person authentication and classification. This file contains a sample subset of EarVN1.0 that consists of selected ear images from male participants (sampled from Person IDs 1–98). The images capture a variety of angles, lighting conditions, and backgrounds to ensure diversity and support robust model training for male ear recognition tasks. [file peerj-cs-10-2603-s002.zip › 075.Rym/075 (91).jpg]

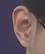

Supplement: Supplemental Information 2 — The EarVN1.0 dataset is a comprehensive collection of over 28,412 ear images from 164 individuals. It encompasses a wide range of variations in pose, scale, illumination, occlusion, resolution, and lighting conditions. This dataset is suitable for various applications such as person authentication and classification. This file contains a sample subset of EarVN1.0 that consists of selected ear images from male participants (sampled from Person IDs 1–98). The images capture a variety of angles, lighting conditions, and backgrounds to ensure diversity and support robust model training for male ear recognition tasks. [file peerj-cs-10-2603-s002.zip › 075.Rym/075 (92).jpg]

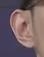

Supplement: Supplemental Information 2 — The EarVN1.0 dataset is a comprehensive collection of over 28,412 ear images from 164 individuals. It encompasses a wide range of variations in pose, scale, illumination, occlusion, resolution, and lighting conditions. This dataset is suitable for various applications such as person authentication and classification. This file contains a sample subset of EarVN1.0 that consists of selected ear images from male participants (sampled from Person IDs 1–98). The images capture a variety of angles, lighting conditions, and backgrounds to ensure diversity and support robust model training for male ear recognition tasks. [file peerj-cs-10-2603-s002.zip › 075.Rym/075 (93).jpg]

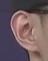

Supplement: Supplemental Information 2 — The EarVN1.0 dataset is a comprehensive collection of over 28,412 ear images from 164 individuals. It encompasses a wide range of variations in pose, scale, illumination, occlusion, resolution, and lighting conditions. This dataset is suitable for various applications such as person authentication and classification. This file contains a sample subset of EarVN1.0 that consists of selected ear images from male participants (sampled from Person IDs 1–98). The images capture a variety of angles, lighting conditions, and backgrounds to ensure diversity and support robust model training for male ear recognition tasks. [file peerj-cs-10-2603-s002.zip › 075.Rym/075 (94).jpg]

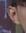

Supplement: Supplemental Information 2 — The EarVN1.0 dataset is a comprehensive collection of over 28,412 ear images from 164 individuals. It encompasses a wide range of variations in pose, scale, illumination, occlusion, resolution, and lighting conditions. This dataset is suitable for various applications such as person authentication and classification. This file contains a sample subset of EarVN1.0 that consists of selected ear images from male participants (sampled from Person IDs 1–98). The images capture a variety of angles, lighting conditions, and backgrounds to ensure diversity and support robust model training for male ear recognition tasks. [file peerj-cs-10-2603-s002.zip › 075.Rym/075 (95).jpg]

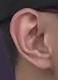

Supplement: Supplemental Information 2 — The EarVN1.0 dataset is a comprehensive collection of over 28,412 ear images from 164 individuals. It encompasses a wide range of variations in pose, scale, illumination, occlusion, resolution, and lighting conditions. This dataset is suitable for various applications such as person authentication and classification. This file contains a sample subset of EarVN1.0 that consists of selected ear images from male participants (sampled from Person IDs 1–98). The images capture a variety of angles, lighting conditions, and backgrounds to ensure diversity and support robust model training for male ear recognition tasks. [file peerj-cs-10-2603-s002.zip › 075.Rym/075 (96).jpg]

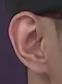

Supplement: Supplemental Information 2 — The EarVN1.0 dataset is a comprehensive collection of over 28,412 ear images from 164 individuals. It encompasses a wide range of variations in pose, scale, illumination, occlusion, resolution, and lighting conditions. This dataset is suitable for various applications such as person authentication and classification. This file contains a sample subset of EarVN1.0 that consists of selected ear images from male participants (sampled from Person IDs 1–98). The images capture a variety of angles, lighting conditions, and backgrounds to ensure diversity and support robust model training for male ear recognition tasks. [file peerj-cs-10-2603-s002.zip › 075.Rym/075 (97).jpg]

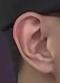

Supplement: Supplemental Information 2 — The EarVN1.0 dataset is a comprehensive collection of over 28,412 ear images from 164 individuals. It encompasses a wide range of variations in pose, scale, illumination, occlusion, resolution, and lighting conditions. This dataset is suitable for various applications such as person authentication and classification. This file contains a sample subset of EarVN1.0 that consists of selected ear images from male participants (sampled from Person IDs 1–98). The images capture a variety of angles, lighting conditions, and backgrounds to ensure diversity and support robust model training for male ear recognition tasks. [file peerj-cs-10-2603-s002.zip › 075.Rym/075 (98).jpg]

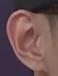

Supplement: Supplemental Information 2 — The EarVN1.0 dataset is a comprehensive collection of over 28,412 ear images from 164 individuals. It encompasses a wide range of variations in pose, scale, illumination, occlusion, resolution, and lighting conditions. This dataset is suitable for various applications such as person authentication and classification. This file contains a sample subset of EarVN1.0 that consists of selected ear images from male participants (sampled from Person IDs 1–98). The images capture a variety of angles, lighting conditions, and backgrounds to ensure diversity and support robust model training for male ear recognition tasks. [file peerj-cs-10-2603-s002.zip › 075.Rym/075 (99).jpg]

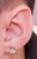

Supplement: Supplemental Information 2 — The EarVN1.0 dataset is a comprehensive collection of over 28,412 ear images from 164 individuals. It encompasses a wide range of variations in pose, scale, illumination, occlusion, resolution, and lighting conditions. This dataset is suitable for various applications such as person authentication and classification. This file contains a sample subset of EarVN1.0 that consists of selected ear images from male participants (sampled from Person IDs 1–98). The images capture a variety of angles, lighting conditions, and backgrounds to ensure diversity and support robust model training for male ear recognition tasks. [file peerj-cs-10-2603-s002.zip › 076.Sky_ST/076 (1).jpg]

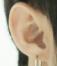

Supplement: Supplemental Information 2 — The EarVN1.0 dataset is a comprehensive collection of over 28,412 ear images from 164 individuals. It encompasses a wide range of variations in pose, scale, illumination, occlusion, resolution, and lighting conditions. This dataset is suitable for various applications such as person authentication and classification. This file contains a sample subset of EarVN1.0 that consists of selected ear images from male participants (sampled from Person IDs 1–98). The images capture a variety of angles, lighting conditions, and backgrounds to ensure diversity and support robust model training for male ear recognition tasks. [file peerj-cs-10-2603-s002.zip › 076.Sky_ST/076 (10).jpg]

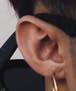

Supplement: Supplemental Information 2 — The EarVN1.0 dataset is a comprehensive collection of over 28,412 ear images from 164 individuals. It encompasses a wide range of variations in pose, scale, illumination, occlusion, resolution, and lighting conditions. This dataset is suitable for various applications such as person authentication and classification. This file contains a sample subset of EarVN1.0 that consists of selected ear images from male participants (sampled from Person IDs 1–98). The images capture a variety of angles, lighting conditions, and backgrounds to ensure diversity and support robust model training for male ear recognition tasks. [file peerj-cs-10-2603-s002.zip › 076.Sky_ST/076 (100).jpg]

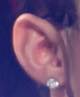

Supplement: Supplemental Information 2 — The EarVN1.0 dataset is a comprehensive collection of over 28,412 ear images from 164 individuals. It encompasses a wide range of variations in pose, scale, illumination, occlusion, resolution, and lighting conditions. This dataset is suitable for various applications such as person authentication and classification. This file contains a sample subset of EarVN1.0 that consists of selected ear images from male participants (sampled from Person IDs 1–98). The images capture a variety of angles, lighting conditions, and backgrounds to ensure diversity and support robust model training for male ear recognition tasks. [file peerj-cs-10-2603-s002.zip › 076.Sky_ST/076 (101).jpg]

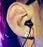

Supplement: Supplemental Information 2 — The EarVN1.0 dataset is a comprehensive collection of over 28,412 ear images from 164 individuals. It encompasses a wide range of variations in pose, scale, illumination, occlusion, resolution, and lighting conditions. This dataset is suitable for various applications such as person authentication and classification. This file contains a sample subset of EarVN1.0 that consists of selected ear images from male participants (sampled from Person IDs 1–98). The images capture a variety of angles, lighting conditions, and backgrounds to ensure diversity and support robust model training for male ear recognition tasks. [file peerj-cs-10-2603-s002.zip › 076.Sky_ST/076 (102).jpg]

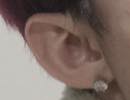

Supplement: Supplemental Information 2 — The EarVN1.0 dataset is a comprehensive collection of over 28,412 ear images from 164 individuals. It encompasses a wide range of variations in pose, scale, illumination, occlusion, resolution, and lighting conditions. This dataset is suitable for various applications such as person authentication and classification. This file contains a sample subset of EarVN1.0 that consists of selected ear images from male participants (sampled from Person IDs 1–98). The images capture a variety of angles, lighting conditions, and backgrounds to ensure diversity and support robust model training for male ear recognition tasks. [file peerj-cs-10-2603-s002.zip › 076.Sky_ST/076 (103).jpg]

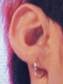

Supplement: Supplemental Information 2 — The EarVN1.0 dataset is a comprehensive collection of over 28,412 ear images from 164 individuals. It encompasses a wide range of variations in pose, scale, illumination, occlusion, resolution, and lighting conditions. This dataset is suitable for various applications such as person authentication and classification. This file contains a sample subset of EarVN1.0 that consists of selected ear images from male participants (sampled from Person IDs 1–98). The images capture a variety of angles, lighting conditions, and backgrounds to ensure diversity and support robust model training for male ear recognition tasks. [file peerj-cs-10-2603-s002.zip › 076.Sky_ST/076 (104).jpg]

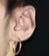

Supplement: Supplemental Information 2 — The EarVN1.0 dataset is a comprehensive collection of over 28,412 ear images from 164 individuals. It encompasses a wide range of variations in pose, scale, illumination, occlusion, resolution, and lighting conditions. This dataset is suitable for various applications such as person authentication and classification. This file contains a sample subset of EarVN1.0 that consists of selected ear images from male participants (sampled from Person IDs 1–98). The images capture a variety of angles, lighting conditions, and backgrounds to ensure diversity and support robust model training for male ear recognition tasks. [file peerj-cs-10-2603-s002.zip › 076.Sky_ST/076 (105).jpg]

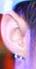

Supplement: Supplemental Information 2 — The EarVN1.0 dataset is a comprehensive collection of over 28,412 ear images from 164 individuals. It encompasses a wide range of variations in pose, scale, illumination, occlusion, resolution, and lighting conditions. This dataset is suitable for various applications such as person authentication and classification. This file contains a sample subset of EarVN1.0 that consists of selected ear images from male participants (sampled from Person IDs 1–98). The images capture a variety of angles, lighting conditions, and backgrounds to ensure diversity and support robust model training for male ear recognition tasks. [file peerj-cs-10-2603-s002.zip › 076.Sky_ST/076 (106).jpg]

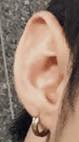

Supplement: Supplemental Information 2 — The EarVN1.0 dataset is a comprehensive collection of over 28,412 ear images from 164 individuals. It encompasses a wide range of variations in pose, scale, illumination, occlusion, resolution, and lighting conditions. This dataset is suitable for various applications such as person authentication and classification. This file contains a sample subset of EarVN1.0 that consists of selected ear images from male participants (sampled from Person IDs 1–98). The images capture a variety of angles, lighting conditions, and backgrounds to ensure diversity and support robust model training for male ear recognition tasks. [file peerj-cs-10-2603-s002.zip › 076.Sky_ST/076 (107).jpg]

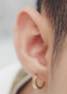

Supplement: Supplemental Information 2 — The EarVN1.0 dataset is a comprehensive collection of over 28,412 ear images from 164 individuals. It encompasses a wide range of variations in pose, scale, illumination, occlusion, resolution, and lighting conditions. This dataset is suitable for various applications such as person authentication and classification. This file contains a sample subset of EarVN1.0 that consists of selected ear images from male participants (sampled from Person IDs 1–98). The images capture a variety of angles, lighting conditions, and backgrounds to ensure diversity and support robust model training for male ear recognition tasks. [file peerj-cs-10-2603-s002.zip › 076.Sky_ST/076 (108).jpg]

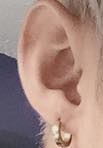

Supplement: Supplemental Information 2 — The EarVN1.0 dataset is a comprehensive collection of over 28,412 ear images from 164 individuals. It encompasses a wide range of variations in pose, scale, illumination, occlusion, resolution, and lighting conditions. This dataset is suitable for various applications such as person authentication and classification. This file contains a sample subset of EarVN1.0 that consists of selected ear images from male participants (sampled from Person IDs 1–98). The images capture a variety of angles, lighting conditions, and backgrounds to ensure diversity and support robust model training for male ear recognition tasks. [file peerj-cs-10-2603-s002.zip › 076.Sky_ST/076 (109).jpg]

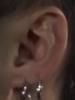

Supplement: Supplemental Information 2 — The EarVN1.0 dataset is a comprehensive collection of over 28,412 ear images from 164 individuals. It encompasses a wide range of variations in pose, scale, illumination, occlusion, resolution, and lighting conditions. This dataset is suitable for various applications such as person authentication and classification. This file contains a sample subset of EarVN1.0 that consists of selected ear images from male participants (sampled from Person IDs 1–98). The images capture a variety of angles, lighting conditions, and backgrounds to ensure diversity and support robust model training for male ear recognition tasks. [file peerj-cs-10-2603-s002.zip › 076.Sky_ST/076 (11).jpg]

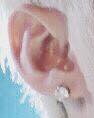

Supplement: Supplemental Information 2 — The EarVN1.0 dataset is a comprehensive collection of over 28,412 ear images from 164 individuals. It encompasses a wide range of variations in pose, scale, illumination, occlusion, resolution, and lighting conditions. This dataset is suitable for various applications such as person authentication and classification. This file contains a sample subset of EarVN1.0 that consists of selected ear images from male participants (sampled from Person IDs 1–98). The images capture a variety of angles, lighting conditions, and backgrounds to ensure diversity and support robust model training for male ear recognition tasks. [file peerj-cs-10-2603-s002.zip › 076.Sky_ST/076 (110).jpg]

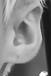

Supplement: Supplemental Information 2 — The EarVN1.0 dataset is a comprehensive collection of over 28,412 ear images from 164 individuals. It encompasses a wide range of variations in pose, scale, illumination, occlusion, resolution, and lighting conditions. This dataset is suitable for various applications such as person authentication and classification. This file contains a sample subset of EarVN1.0 that consists of selected ear images from male participants (sampled from Person IDs 1–98). The images capture a variety of angles, lighting conditions, and backgrounds to ensure diversity and support robust model training for male ear recognition tasks. [file peerj-cs-10-2603-s002.zip › 076.Sky_ST/076 (111).jpg]

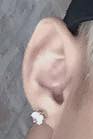

Supplement: Supplemental Information 2 — The EarVN1.0 dataset is a comprehensive collection of over 28,412 ear images from 164 individuals. It encompasses a wide range of variations in pose, scale, illumination, occlusion, resolution, and lighting conditions. This dataset is suitable for various applications such as person authentication and classification. This file contains a sample subset of EarVN1.0 that consists of selected ear images from male participants (sampled from Person IDs 1–98). The images capture a variety of angles, lighting conditions, and backgrounds to ensure diversity and support robust model training for male ear recognition tasks. [file peerj-cs-10-2603-s002.zip › 076.Sky_ST/076 (112).jpg]

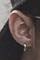

Supplement: Supplemental Information 2 — The EarVN1.0 dataset is a comprehensive collection of over 28,412 ear images from 164 individuals. It encompasses a wide range of variations in pose, scale, illumination, occlusion, resolution, and lighting conditions. This dataset is suitable for various applications such as person authentication and classification. This file contains a sample subset of EarVN1.0 that consists of selected ear images from male participants (sampled from Person IDs 1–98). The images capture a variety of angles, lighting conditions, and backgrounds to ensure diversity and support robust model training for male ear recognition tasks. [file peerj-cs-10-2603-s002.zip › 076.Sky_ST/076 (113).jpg]

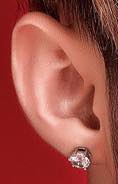

Supplement: Supplemental Information 2 — The EarVN1.0 dataset is a comprehensive collection of over 28,412 ear images from 164 individuals. It encompasses a wide range of variations in pose, scale, illumination, occlusion, resolution, and lighting conditions. This dataset is suitable for various applications such as person authentication and classification. This file contains a sample subset of EarVN1.0 that consists of selected ear images from male participants (sampled from Person IDs 1–98). The images capture a variety of angles, lighting conditions, and backgrounds to ensure diversity and support robust model training for male ear recognition tasks. [file peerj-cs-10-2603-s002.zip › 076.Sky_ST/076 (114).jpg]

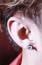

Supplement: Supplemental Information 2 — The EarVN1.0 dataset is a comprehensive collection of over 28,412 ear images from 164 individuals. It encompasses a wide range of variations in pose, scale, illumination, occlusion, resolution, and lighting conditions. This dataset is suitable for various applications such as person authentication and classification. This file contains a sample subset of EarVN1.0 that consists of selected ear images from male participants (sampled from Person IDs 1–98). The images capture a variety of angles, lighting conditions, and backgrounds to ensure diversity and support robust model training for male ear recognition tasks. [file peerj-cs-10-2603-s002.zip › 076.Sky_ST/076 (115).jpg]

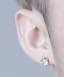

Supplement: Supplemental Information 2 — The EarVN1.0 dataset is a comprehensive collection of over 28,412 ear images from 164 individuals. It encompasses a wide range of variations in pose, scale, illumination, occlusion, resolution, and lighting conditions. This dataset is suitable for various applications such as person authentication and classification. This file contains a sample subset of EarVN1.0 that consists of selected ear images from male participants (sampled from Person IDs 1–98). The images capture a variety of angles, lighting conditions, and backgrounds to ensure diversity and support robust model training for male ear recognition tasks. [file peerj-cs-10-2603-s002.zip › 076.Sky_ST/076 (116).jpg]

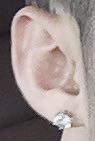

Supplement: Supplemental Information 2 — The EarVN1.0 dataset is a comprehensive collection of over 28,412 ear images from 164 individuals. It encompasses a wide range of variations in pose, scale, illumination, occlusion, resolution, and lighting conditions. This dataset is suitable for various applications such as person authentication and classification. This file contains a sample subset of EarVN1.0 that consists of selected ear images from male participants (sampled from Person IDs 1–98). The images capture a variety of angles, lighting conditions, and backgrounds to ensure diversity and support robust model training for male ear recognition tasks. [file peerj-cs-10-2603-s002.zip › 076.Sky_ST/076 (117).jpg]

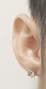

Supplement: Supplemental Information 2 — The EarVN1.0 dataset is a comprehensive collection of over 28,412 ear images from 164 individuals. It encompasses a wide range of variations in pose, scale, illumination, occlusion, resolution, and lighting conditions. This dataset is suitable for various applications such as person authentication and classification. This file contains a sample subset of EarVN1.0 that consists of selected ear images from male participants (sampled from Person IDs 1–98). The images capture a variety of angles, lighting conditions, and backgrounds to ensure diversity and support robust model training for male ear recognition tasks. [file peerj-cs-10-2603-s002.zip › 076.Sky_ST/076 (118).jpg]

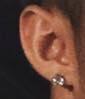

Supplement: Supplemental Information 2 — The EarVN1.0 dataset is a comprehensive collection of over 28,412 ear images from 164 individuals. It encompasses a wide range of variations in pose, scale, illumination, occlusion, resolution, and lighting conditions. This dataset is suitable for various applications such as person authentication and classification. This file contains a sample subset of EarVN1.0 that consists of selected ear images from male participants (sampled from Person IDs 1–98). The images capture a variety of angles, lighting conditions, and backgrounds to ensure diversity and support robust model training for male ear recognition tasks. [file peerj-cs-10-2603-s002.zip › 076.Sky_ST/076 (119).jpg]

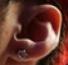

Supplement: Supplemental Information 2 — The EarVN1.0 dataset is a comprehensive collection of over 28,412 ear images from 164 individuals. It encompasses a wide range of variations in pose, scale, illumination, occlusion, resolution, and lighting conditions. This dataset is suitable for various applications such as person authentication and classification. This file contains a sample subset of EarVN1.0 that consists of selected ear images from male participants (sampled from Person IDs 1–98). The images capture a variety of angles, lighting conditions, and backgrounds to ensure diversity and support robust model training for male ear recognition tasks. [file peerj-cs-10-2603-s002.zip › 076.Sky_ST/076 (12).jpg]

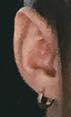

Supplement: Supplemental Information 2 — The EarVN1.0 dataset is a comprehensive collection of over 28,412 ear images from 164 individuals. It encompasses a wide range of variations in pose, scale, illumination, occlusion, resolution, and lighting conditions. This dataset is suitable for various applications such as person authentication and classification. This file contains a sample subset of EarVN1.0 that consists of selected ear images from male participants (sampled from Person IDs 1–98). The images capture a variety of angles, lighting conditions, and backgrounds to ensure diversity and support robust model training for male ear recognition tasks. [file peerj-cs-10-2603-s002.zip › 076.Sky_ST/076 (120).jpg]

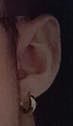

Supplement: Supplemental Information 2 — The EarVN1.0 dataset is a comprehensive collection of over 28,412 ear images from 164 individuals. It encompasses a wide range of variations in pose, scale, illumination, occlusion, resolution, and lighting conditions. This dataset is suitable for various applications such as person authentication and classification. This file contains a sample subset of EarVN1.0 that consists of selected ear images from male participants (sampled from Person IDs 1–98). The images capture a variety of angles, lighting conditions, and backgrounds to ensure diversity and support robust model training for male ear recognition tasks. [file peerj-cs-10-2603-s002.zip › 076.Sky_ST/076 (121).jpg]

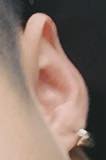

Supplement: Supplemental Information 2 — The EarVN1.0 dataset is a comprehensive collection of over 28,412 ear images from 164 individuals. It encompasses a wide range of variations in pose, scale, illumination, occlusion, resolution, and lighting conditions. This dataset is suitable for various applications such as person authentication and classification. This file contains a sample subset of EarVN1.0 that consists of selected ear images from male participants (sampled from Person IDs 1–98). The images capture a variety of angles, lighting conditions, and backgrounds to ensure diversity and support robust model training for male ear recognition tasks. [file peerj-cs-10-2603-s002.zip › 076.Sky_ST/076 (122).jpg]

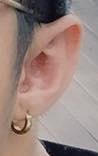

Supplement: Supplemental Information 2 — The EarVN1.0 dataset is a comprehensive collection of over 28,412 ear images from 164 individuals. It encompasses a wide range of variations in pose, scale, illumination, occlusion, resolution, and lighting conditions. This dataset is suitable for various applications such as person authentication and classification. This file contains a sample subset of EarVN1.0 that consists of selected ear images from male participants (sampled from Person IDs 1–98). The images capture a variety of angles, lighting conditions, and backgrounds to ensure diversity and support robust model training for male ear recognition tasks. [file peerj-cs-10-2603-s002.zip › 076.Sky_ST/076 (123).jpg]

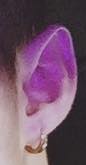

Supplement: Supplemental Information 2 — The EarVN1.0 dataset is a comprehensive collection of over 28,412 ear images from 164 individuals. It encompasses a wide range of variations in pose, scale, illumination, occlusion, resolution, and lighting conditions. This dataset is suitable for various applications such as person authentication and classification. This file contains a sample subset of EarVN1.0 that consists of selected ear images from male participants (sampled from Person IDs 1–98). The images capture a variety of angles, lighting conditions, and backgrounds to ensure diversity and support robust model training for male ear recognition tasks. [file peerj-cs-10-2603-s002.zip › 076.Sky_ST/076 (124).jpg]

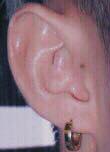

Supplement: Supplemental Information 2 — The EarVN1.0 dataset is a comprehensive collection of over 28,412 ear images from 164 individuals. It encompasses a wide range of variations in pose, scale, illumination, occlusion, resolution, and lighting conditions. This dataset is suitable for various applications such as person authentication and classification. This file contains a sample subset of EarVN1.0 that consists of selected ear images from male participants (sampled from Person IDs 1–98). The images capture a variety of angles, lighting conditions, and backgrounds to ensure diversity and support robust model training for male ear recognition tasks. [file peerj-cs-10-2603-s002.zip › 076.Sky_ST/076 (125).jpg]

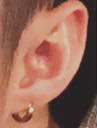

Supplement: Supplemental Information 2 — The EarVN1.0 dataset is a comprehensive collection of over 28,412 ear images from 164 individuals. It encompasses a wide range of variations in pose, scale, illumination, occlusion, resolution, and lighting conditions. This dataset is suitable for various applications such as person authentication and classification. This file contains a sample subset of EarVN1.0 that consists of selected ear images from male participants (sampled from Person IDs 1–98). The images capture a variety of angles, lighting conditions, and backgrounds to ensure diversity and support robust model training for male ear recognition tasks. [file peerj-cs-10-2603-s002.zip › 076.Sky_ST/076 (126).jpg]

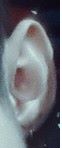

Supplement: Supplemental Information 2 — The EarVN1.0 dataset is a comprehensive collection of over 28,412 ear images from 164 individuals. It encompasses a wide range of variations in pose, scale, illumination, occlusion, resolution, and lighting conditions. This dataset is suitable for various applications such as person authentication and classification. This file contains a sample subset of EarVN1.0 that consists of selected ear images from male participants (sampled from Person IDs 1–98). The images capture a variety of angles, lighting conditions, and backgrounds to ensure diversity and support robust model training for male ear recognition tasks. [file peerj-cs-10-2603-s002.zip › 076.Sky_ST/076 (127).jpg]

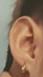

Supplement: Supplemental Information 2 — The EarVN1.0 dataset is a comprehensive collection of over 28,412 ear images from 164 individuals. It encompasses a wide range of variations in pose, scale, illumination, occlusion, resolution, and lighting conditions. This dataset is suitable for various applications such as person authentication and classification. This file contains a sample subset of EarVN1.0 that consists of selected ear images from male participants (sampled from Person IDs 1–98). The images capture a variety of angles, lighting conditions, and backgrounds to ensure diversity and support robust model training for male ear recognition tasks. [file peerj-cs-10-2603-s002.zip › 076.Sky_ST/076 (128).jpg]

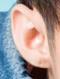

Supplement: Supplemental Information 2 — The EarVN1.0 dataset is a comprehensive collection of over 28,412 ear images from 164 individuals. It encompasses a wide range of variations in pose, scale, illumination, occlusion, resolution, and lighting conditions. This dataset is suitable for various applications such as person authentication and classification. This file contains a sample subset of EarVN1.0 that consists of selected ear images from male participants (sampled from Person IDs 1–98). The images capture a variety of angles, lighting conditions, and backgrounds to ensure diversity and support robust model training for male ear recognition tasks. [file peerj-cs-10-2603-s002.zip › 076.Sky_ST/076 (129).jpg]

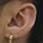

Supplement: Supplemental Information 2 — The EarVN1.0 dataset is a comprehensive collection of over 28,412 ear images from 164 individuals. It encompasses a wide range of variations in pose, scale, illumination, occlusion, resolution, and lighting conditions. This dataset is suitable for various applications such as person authentication and classification. This file contains a sample subset of EarVN1.0 that consists of selected ear images from male participants (sampled from Person IDs 1–98). The images capture a variety of angles, lighting conditions, and backgrounds to ensure diversity and support robust model training for male ear recognition tasks. [file peerj-cs-10-2603-s002.zip › 076.Sky_ST/076 (13).jpg]

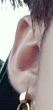

Supplement: Supplemental Information 2 — The EarVN1.0 dataset is a comprehensive collection of over 28,412 ear images from 164 individuals. It encompasses a wide range of variations in pose, scale, illumination, occlusion, resolution, and lighting conditions. This dataset is suitable for various applications such as person authentication and classification. This file contains a sample subset of EarVN1.0 that consists of selected ear images from male participants (sampled from Person IDs 1–98). The images capture a variety of angles, lighting conditions, and backgrounds to ensure diversity and support robust model training for male ear recognition tasks. [file peerj-cs-10-2603-s002.zip › 076.Sky_ST/076 (130).jpg]

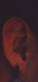

Supplement: Supplemental Information 2 — The EarVN1.0 dataset is a comprehensive collection of over 28,412 ear images from 164 individuals. It encompasses a wide range of variations in pose, scale, illumination, occlusion, resolution, and lighting conditions. This dataset is suitable for various applications such as person authentication and classification. This file contains a sample subset of EarVN1.0 that consists of selected ear images from male participants (sampled from Person IDs 1–98). The images capture a variety of angles, lighting conditions, and backgrounds to ensure diversity and support robust model training for male ear recognition tasks. [file peerj-cs-10-2603-s002.zip › 076.Sky_ST/076 (131).jpg]

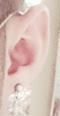

Supplement: Supplemental Information 2 — The EarVN1.0 dataset is a comprehensive collection of over 28,412 ear images from 164 individuals. It encompasses a wide range of variations in pose, scale, illumination, occlusion, resolution, and lighting conditions. This dataset is suitable for various applications such as person authentication and classification. This file contains a sample subset of EarVN1.0 that consists of selected ear images from male participants (sampled from Person IDs 1–98). The images capture a variety of angles, lighting conditions, and backgrounds to ensure diversity and support robust model training for male ear recognition tasks. [file peerj-cs-10-2603-s002.zip › 076.Sky_ST/076 (132).jpg]

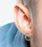

Supplement: Supplemental Information 2 — The EarVN1.0 dataset is a comprehensive collection of over 28,412 ear images from 164 individuals. It encompasses a wide range of variations in pose, scale, illumination, occlusion, resolution, and lighting conditions. This dataset is suitable for various applications such as person authentication and classification. This file contains a sample subset of EarVN1.0 that consists of selected ear images from male participants (sampled from Person IDs 1–98). The images capture a variety of angles, lighting conditions, and backgrounds to ensure diversity and support robust model training for male ear recognition tasks. [file peerj-cs-10-2603-s002.zip › 076.Sky_ST/076 (133).jpg]

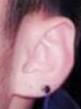

Supplement: Supplemental Information 2 — The EarVN1.0 dataset is a comprehensive collection of over 28,412 ear images from 164 individuals. It encompasses a wide range of variations in pose, scale, illumination, occlusion, resolution, and lighting conditions. This dataset is suitable for various applications such as person authentication and classification. This file contains a sample subset of EarVN1.0 that consists of selected ear images from male participants (sampled from Person IDs 1–98). The images capture a variety of angles, lighting conditions, and backgrounds to ensure diversity and support robust model training for male ear recognition tasks. [file peerj-cs-10-2603-s002.zip › 076.Sky_ST/076 (134).jpg]
