# Supplementary figures and images for: DANNET: deep attention neural network for efficient ear identification in biometrics (part 1 of 4)
Source: PeerJ Comput Sci. 2024 Dec 18;10:e2603. doi: 10.7717/peerj-cs.2603 (PMC11784740; doi:10.7717/peerj-cs.2603)

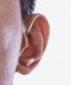

Supplement: Supplemental Information 2 — The EarVN1.0 dataset is a comprehensive collection of over 28,412 ear images from 164 individuals. It encompasses a wide range of variations in pose, scale, illumination, occlusion, resolution, and lighting conditions. This dataset is suitable for various applications such as person authentication and classification. This file contains a sample subset of EarVN1.0 that consists of selected ear images from male participants (sampled from Person IDs 1–98). The images capture a variety of angles, lighting conditions, and backgrounds to ensure diversity and support robust model training for male ear recognition tasks. [file peerj-cs-10-2603-s002.zip › 075.Rym/075 (1).jpg]

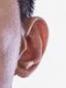

Supplement: Supplemental Information 2 — The EarVN1.0 dataset is a comprehensive collection of over 28,412 ear images from 164 individuals. It encompasses a wide range of variations in pose, scale, illumination, occlusion, resolution, and lighting conditions. This dataset is suitable for various applications such as person authentication and classification. This file contains a sample subset of EarVN1.0 that consists of selected ear images from male participants (sampled from Person IDs 1–98). The images capture a variety of angles, lighting conditions, and backgrounds to ensure diversity and support robust model training for male ear recognition tasks. [file peerj-cs-10-2603-s002.zip › 075.Rym/075 (10).jpg]

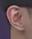

Supplement: Supplemental Information 2 — The EarVN1.0 dataset is a comprehensive collection of over 28,412 ear images from 164 individuals. It encompasses a wide range of variations in pose, scale, illumination, occlusion, resolution, and lighting conditions. This dataset is suitable for various applications such as person authentication and classification. This file contains a sample subset of EarVN1.0 that consists of selected ear images from male participants (sampled from Person IDs 1–98). The images capture a variety of angles, lighting conditions, and backgrounds to ensure diversity and support robust model training for male ear recognition tasks. [file peerj-cs-10-2603-s002.zip › 075.Rym/075 (100).jpg]

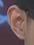

Supplement: Supplemental Information 2 — The EarVN1.0 dataset is a comprehensive collection of over 28,412 ear images from 164 individuals. It encompasses a wide range of variations in pose, scale, illumination, occlusion, resolution, and lighting conditions. This dataset is suitable for various applications such as person authentication and classification. This file contains a sample subset of EarVN1.0 that consists of selected ear images from male participants (sampled from Person IDs 1–98). The images capture a variety of angles, lighting conditions, and backgrounds to ensure diversity and support robust model training for male ear recognition tasks. [file peerj-cs-10-2603-s002.zip › 075.Rym/075 (101).jpg]

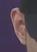

Supplement: Supplemental Information 2 — The EarVN1.0 dataset is a comprehensive collection of over 28,412 ear images from 164 individuals. It encompasses a wide range of variations in pose, scale, illumination, occlusion, resolution, and lighting conditions. This dataset is suitable for various applications such as person authentication and classification. This file contains a sample subset of EarVN1.0 that consists of selected ear images from male participants (sampled from Person IDs 1–98). The images capture a variety of angles, lighting conditions, and backgrounds to ensure diversity and support robust model training for male ear recognition tasks. [file peerj-cs-10-2603-s002.zip › 075.Rym/075 (102).jpg]

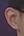

Supplement: Supplemental Information 2 — The EarVN1.0 dataset is a comprehensive collection of over 28,412 ear images from 164 individuals. It encompasses a wide range of variations in pose, scale, illumination, occlusion, resolution, and lighting conditions. This dataset is suitable for various applications such as person authentication and classification. This file contains a sample subset of EarVN1.0 that consists of selected ear images from male participants (sampled from Person IDs 1–98). The images capture a variety of angles, lighting conditions, and backgrounds to ensure diversity and support robust model training for male ear recognition tasks. [file peerj-cs-10-2603-s002.zip › 075.Rym/075 (103).jpg]

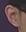

Supplement: Supplemental Information 2 — The EarVN1.0 dataset is a comprehensive collection of over 28,412 ear images from 164 individuals. It encompasses a wide range of variations in pose, scale, illumination, occlusion, resolution, and lighting conditions. This dataset is suitable for various applications such as person authentication and classification. This file contains a sample subset of EarVN1.0 that consists of selected ear images from male participants (sampled from Person IDs 1–98). The images capture a variety of angles, lighting conditions, and backgrounds to ensure diversity and support robust model training for male ear recognition tasks. [file peerj-cs-10-2603-s002.zip › 075.Rym/075 (104).jpg]

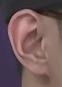

Supplement: Supplemental Information 2 — The EarVN1.0 dataset is a comprehensive collection of over 28,412 ear images from 164 individuals. It encompasses a wide range of variations in pose, scale, illumination, occlusion, resolution, and lighting conditions. This dataset is suitable for various applications such as person authentication and classification. This file contains a sample subset of EarVN1.0 that consists of selected ear images from male participants (sampled from Person IDs 1–98). The images capture a variety of angles, lighting conditions, and backgrounds to ensure diversity and support robust model training for male ear recognition tasks. [file peerj-cs-10-2603-s002.zip › 075.Rym/075 (105).jpg]

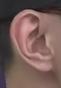

Supplement: Supplemental Information 2 — The EarVN1.0 dataset is a comprehensive collection of over 28,412 ear images from 164 individuals. It encompasses a wide range of variations in pose, scale, illumination, occlusion, resolution, and lighting conditions. This dataset is suitable for various applications such as person authentication and classification. This file contains a sample subset of EarVN1.0 that consists of selected ear images from male participants (sampled from Person IDs 1–98). The images capture a variety of angles, lighting conditions, and backgrounds to ensure diversity and support robust model training for male ear recognition tasks. [file peerj-cs-10-2603-s002.zip › 075.Rym/075 (106).jpg]

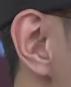

Supplement: Supplemental Information 2 — The EarVN1.0 dataset is a comprehensive collection of over 28,412 ear images from 164 individuals. It encompasses a wide range of variations in pose, scale, illumination, occlusion, resolution, and lighting conditions. This dataset is suitable for various applications such as person authentication and classification. This file contains a sample subset of EarVN1.0 that consists of selected ear images from male participants (sampled from Person IDs 1–98). The images capture a variety of angles, lighting conditions, and backgrounds to ensure diversity and support robust model training for male ear recognition tasks. [file peerj-cs-10-2603-s002.zip › 075.Rym/075 (107).jpg]

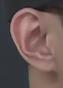

Supplement: Supplemental Information 2 — The EarVN1.0 dataset is a comprehensive collection of over 28,412 ear images from 164 individuals. It encompasses a wide range of variations in pose, scale, illumination, occlusion, resolution, and lighting conditions. This dataset is suitable for various applications such as person authentication and classification. This file contains a sample subset of EarVN1.0 that consists of selected ear images from male participants (sampled from Person IDs 1–98). The images capture a variety of angles, lighting conditions, and backgrounds to ensure diversity and support robust model training for male ear recognition tasks. [file peerj-cs-10-2603-s002.zip › 075.Rym/075 (108).jpg]

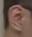

Supplement: Supplemental Information 2 — The EarVN1.0 dataset is a comprehensive collection of over 28,412 ear images from 164 individuals. It encompasses a wide range of variations in pose, scale, illumination, occlusion, resolution, and lighting conditions. This dataset is suitable for various applications such as person authentication and classification. This file contains a sample subset of EarVN1.0 that consists of selected ear images from male participants (sampled from Person IDs 1–98). The images capture a variety of angles, lighting conditions, and backgrounds to ensure diversity and support robust model training for male ear recognition tasks. [file peerj-cs-10-2603-s002.zip › 075.Rym/075 (109).jpg]

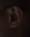

Supplement: Supplemental Information 2 — The EarVN1.0 dataset is a comprehensive collection of over 28,412 ear images from 164 individuals. It encompasses a wide range of variations in pose, scale, illumination, occlusion, resolution, and lighting conditions. This dataset is suitable for various applications such as person authentication and classification. This file contains a sample subset of EarVN1.0 that consists of selected ear images from male participants (sampled from Person IDs 1–98). The images capture a variety of angles, lighting conditions, and backgrounds to ensure diversity and support robust model training for male ear recognition tasks. [file peerj-cs-10-2603-s002.zip › 075.Rym/075 (11).jpg]

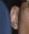

Supplement: Supplemental Information 2 — The EarVN1.0 dataset is a comprehensive collection of over 28,412 ear images from 164 individuals. It encompasses a wide range of variations in pose, scale, illumination, occlusion, resolution, and lighting conditions. This dataset is suitable for various applications such as person authentication and classification. This file contains a sample subset of EarVN1.0 that consists of selected ear images from male participants (sampled from Person IDs 1–98). The images capture a variety of angles, lighting conditions, and backgrounds to ensure diversity and support robust model training for male ear recognition tasks. [file peerj-cs-10-2603-s002.zip › 075.Rym/075 (110).jpg]

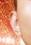

Supplement: Supplemental Information 2 — The EarVN1.0 dataset is a comprehensive collection of over 28,412 ear images from 164 individuals. It encompasses a wide range of variations in pose, scale, illumination, occlusion, resolution, and lighting conditions. This dataset is suitable for various applications such as person authentication and classification. This file contains a sample subset of EarVN1.0 that consists of selected ear images from male participants (sampled from Person IDs 1–98). The images capture a variety of angles, lighting conditions, and backgrounds to ensure diversity and support robust model training for male ear recognition tasks. [file peerj-cs-10-2603-s002.zip › 075.Rym/075 (111).jpg]

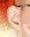

Supplement: Supplemental Information 2 — The EarVN1.0 dataset is a comprehensive collection of over 28,412 ear images from 164 individuals. It encompasses a wide range of variations in pose, scale, illumination, occlusion, resolution, and lighting conditions. This dataset is suitable for various applications such as person authentication and classification. This file contains a sample subset of EarVN1.0 that consists of selected ear images from male participants (sampled from Person IDs 1–98). The images capture a variety of angles, lighting conditions, and backgrounds to ensure diversity and support robust model training for male ear recognition tasks. [file peerj-cs-10-2603-s002.zip › 075.Rym/075 (112).jpg]

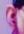

Supplement: Supplemental Information 2 — The EarVN1.0 dataset is a comprehensive collection of over 28,412 ear images from 164 individuals. It encompasses a wide range of variations in pose, scale, illumination, occlusion, resolution, and lighting conditions. This dataset is suitable for various applications such as person authentication and classification. This file contains a sample subset of EarVN1.0 that consists of selected ear images from male participants (sampled from Person IDs 1–98). The images capture a variety of angles, lighting conditions, and backgrounds to ensure diversity and support robust model training for male ear recognition tasks. [file peerj-cs-10-2603-s002.zip › 075.Rym/075 (113).jpg]

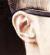

Supplement: Supplemental Information 2 — The EarVN1.0 dataset is a comprehensive collection of over 28,412 ear images from 164 individuals. It encompasses a wide range of variations in pose, scale, illumination, occlusion, resolution, and lighting conditions. This dataset is suitable for various applications such as person authentication and classification. This file contains a sample subset of EarVN1.0 that consists of selected ear images from male participants (sampled from Person IDs 1–98). The images capture a variety of angles, lighting conditions, and backgrounds to ensure diversity and support robust model training for male ear recognition tasks. [file peerj-cs-10-2603-s002.zip › 075.Rym/075 (114).jpg]

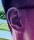

Supplement: Supplemental Information 2 — The EarVN1.0 dataset is a comprehensive collection of over 28,412 ear images from 164 individuals. It encompasses a wide range of variations in pose, scale, illumination, occlusion, resolution, and lighting conditions. This dataset is suitable for various applications such as person authentication and classification. This file contains a sample subset of EarVN1.0 that consists of selected ear images from male participants (sampled from Person IDs 1–98). The images capture a variety of angles, lighting conditions, and backgrounds to ensure diversity and support robust model training for male ear recognition tasks. [file peerj-cs-10-2603-s002.zip › 075.Rym/075 (115).jpg]

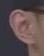

Supplement: Supplemental Information 2 — The EarVN1.0 dataset is a comprehensive collection of over 28,412 ear images from 164 individuals. It encompasses a wide range of variations in pose, scale, illumination, occlusion, resolution, and lighting conditions. This dataset is suitable for various applications such as person authentication and classification. This file contains a sample subset of EarVN1.0 that consists of selected ear images from male participants (sampled from Person IDs 1–98). The images capture a variety of angles, lighting conditions, and backgrounds to ensure diversity and support robust model training for male ear recognition tasks. [file peerj-cs-10-2603-s002.zip › 075.Rym/075 (116).jpg]

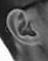

Supplement: Supplemental Information 2 — The EarVN1.0 dataset is a comprehensive collection of over 28,412 ear images from 164 individuals. It encompasses a wide range of variations in pose, scale, illumination, occlusion, resolution, and lighting conditions. This dataset is suitable for various applications such as person authentication and classification. This file contains a sample subset of EarVN1.0 that consists of selected ear images from male participants (sampled from Person IDs 1–98). The images capture a variety of angles, lighting conditions, and backgrounds to ensure diversity and support robust model training for male ear recognition tasks. [file peerj-cs-10-2603-s002.zip › 075.Rym/075 (117).jpg]

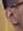

Supplement: Supplemental Information 2 — The EarVN1.0 dataset is a comprehensive collection of over 28,412 ear images from 164 individuals. It encompasses a wide range of variations in pose, scale, illumination, occlusion, resolution, and lighting conditions. This dataset is suitable for various applications such as person authentication and classification. This file contains a sample subset of EarVN1.0 that consists of selected ear images from male participants (sampled from Person IDs 1–98). The images capture a variety of angles, lighting conditions, and backgrounds to ensure diversity and support robust model training for male ear recognition tasks. [file peerj-cs-10-2603-s002.zip › 075.Rym/075 (118).jpg]

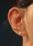

Supplement: Supplemental Information 2 — The EarVN1.0 dataset is a comprehensive collection of over 28,412 ear images from 164 individuals. It encompasses a wide range of variations in pose, scale, illumination, occlusion, resolution, and lighting conditions. This dataset is suitable for various applications such as person authentication and classification. This file contains a sample subset of EarVN1.0 that consists of selected ear images from male participants (sampled from Person IDs 1–98). The images capture a variety of angles, lighting conditions, and backgrounds to ensure diversity and support robust model training for male ear recognition tasks. [file peerj-cs-10-2603-s002.zip › 075.Rym/075 (119).jpg]

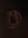

Supplement: Supplemental Information 2 — The EarVN1.0 dataset is a comprehensive collection of over 28,412 ear images from 164 individuals. It encompasses a wide range of variations in pose, scale, illumination, occlusion, resolution, and lighting conditions. This dataset is suitable for various applications such as person authentication and classification. This file contains a sample subset of EarVN1.0 that consists of selected ear images from male participants (sampled from Person IDs 1–98). The images capture a variety of angles, lighting conditions, and backgrounds to ensure diversity and support robust model training for male ear recognition tasks. [file peerj-cs-10-2603-s002.zip › 075.Rym/075 (12).jpg]

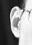

Supplement: Supplemental Information 2 — The EarVN1.0 dataset is a comprehensive collection of over 28,412 ear images from 164 individuals. It encompasses a wide range of variations in pose, scale, illumination, occlusion, resolution, and lighting conditions. This dataset is suitable for various applications such as person authentication and classification. This file contains a sample subset of EarVN1.0 that consists of selected ear images from male participants (sampled from Person IDs 1–98). The images capture a variety of angles, lighting conditions, and backgrounds to ensure diversity and support robust model training for male ear recognition tasks. [file peerj-cs-10-2603-s002.zip › 075.Rym/075 (120).jpg]

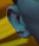

Supplement: Supplemental Information 2 — The EarVN1.0 dataset is a comprehensive collection of over 28,412 ear images from 164 individuals. It encompasses a wide range of variations in pose, scale, illumination, occlusion, resolution, and lighting conditions. This dataset is suitable for various applications such as person authentication and classification. This file contains a sample subset of EarVN1.0 that consists of selected ear images from male participants (sampled from Person IDs 1–98). The images capture a variety of angles, lighting conditions, and backgrounds to ensure diversity and support robust model training for male ear recognition tasks. [file peerj-cs-10-2603-s002.zip › 075.Rym/075 (121).jpg]

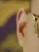

Supplement: Supplemental Information 2 — The EarVN1.0 dataset is a comprehensive collection of over 28,412 ear images from 164 individuals. It encompasses a wide range of variations in pose, scale, illumination, occlusion, resolution, and lighting conditions. This dataset is suitable for various applications such as person authentication and classification. This file contains a sample subset of EarVN1.0 that consists of selected ear images from male participants (sampled from Person IDs 1–98). The images capture a variety of angles, lighting conditions, and backgrounds to ensure diversity and support robust model training for male ear recognition tasks. [file peerj-cs-10-2603-s002.zip › 075.Rym/075 (122).jpg]

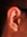

Supplement: Supplemental Information 2 — The EarVN1.0 dataset is a comprehensive collection of over 28,412 ear images from 164 individuals. It encompasses a wide range of variations in pose, scale, illumination, occlusion, resolution, and lighting conditions. This dataset is suitable for various applications such as person authentication and classification. This file contains a sample subset of EarVN1.0 that consists of selected ear images from male participants (sampled from Person IDs 1–98). The images capture a variety of angles, lighting conditions, and backgrounds to ensure diversity and support robust model training for male ear recognition tasks. [file peerj-cs-10-2603-s002.zip › 075.Rym/075 (123).jpg]

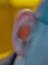

Supplement: Supplemental Information 2 — The EarVN1.0 dataset is a comprehensive collection of over 28,412 ear images from 164 individuals. It encompasses a wide range of variations in pose, scale, illumination, occlusion, resolution, and lighting conditions. This dataset is suitable for various applications such as person authentication and classification. This file contains a sample subset of EarVN1.0 that consists of selected ear images from male participants (sampled from Person IDs 1–98). The images capture a variety of angles, lighting conditions, and backgrounds to ensure diversity and support robust model training for male ear recognition tasks. [file peerj-cs-10-2603-s002.zip › 075.Rym/075 (124).jpg]

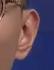

Supplement: Supplemental Information 2 — The EarVN1.0 dataset is a comprehensive collection of over 28,412 ear images from 164 individuals. It encompasses a wide range of variations in pose, scale, illumination, occlusion, resolution, and lighting conditions. This dataset is suitable for various applications such as person authentication and classification. This file contains a sample subset of EarVN1.0 that consists of selected ear images from male participants (sampled from Person IDs 1–98). The images capture a variety of angles, lighting conditions, and backgrounds to ensure diversity and support robust model training for male ear recognition tasks. [file peerj-cs-10-2603-s002.zip › 075.Rym/075 (125).jpg]

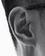

Supplement: Supplemental Information 2 — The EarVN1.0 dataset is a comprehensive collection of over 28,412 ear images from 164 individuals. It encompasses a wide range of variations in pose, scale, illumination, occlusion, resolution, and lighting conditions. This dataset is suitable for various applications such as person authentication and classification. This file contains a sample subset of EarVN1.0 that consists of selected ear images from male participants (sampled from Person IDs 1–98). The images capture a variety of angles, lighting conditions, and backgrounds to ensure diversity and support robust model training for male ear recognition tasks. [file peerj-cs-10-2603-s002.zip › 075.Rym/075 (126).jpg]

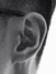

Supplement: Supplemental Information 2 — The EarVN1.0 dataset is a comprehensive collection of over 28,412 ear images from 164 individuals. It encompasses a wide range of variations in pose, scale, illumination, occlusion, resolution, and lighting conditions. This dataset is suitable for various applications such as person authentication and classification. This file contains a sample subset of EarVN1.0 that consists of selected ear images from male participants (sampled from Person IDs 1–98). The images capture a variety of angles, lighting conditions, and backgrounds to ensure diversity and support robust model training for male ear recognition tasks. [file peerj-cs-10-2603-s002.zip › 075.Rym/075 (127).jpg]

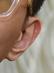

Supplement: Supplemental Information 2 — The EarVN1.0 dataset is a comprehensive collection of over 28,412 ear images from 164 individuals. It encompasses a wide range of variations in pose, scale, illumination, occlusion, resolution, and lighting conditions. This dataset is suitable for various applications such as person authentication and classification. This file contains a sample subset of EarVN1.0 that consists of selected ear images from male participants (sampled from Person IDs 1–98). The images capture a variety of angles, lighting conditions, and backgrounds to ensure diversity and support robust model training for male ear recognition tasks. [file peerj-cs-10-2603-s002.zip › 075.Rym/075 (128).jpg]

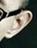

Supplement: Supplemental Information 2 — The EarVN1.0 dataset is a comprehensive collection of over 28,412 ear images from 164 individuals. It encompasses a wide range of variations in pose, scale, illumination, occlusion, resolution, and lighting conditions. This dataset is suitable for various applications such as person authentication and classification. This file contains a sample subset of EarVN1.0 that consists of selected ear images from male participants (sampled from Person IDs 1–98). The images capture a variety of angles, lighting conditions, and backgrounds to ensure diversity and support robust model training for male ear recognition tasks. [file peerj-cs-10-2603-s002.zip › 075.Rym/075 (129).jpg]

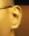

Supplement: Supplemental Information 2 — The EarVN1.0 dataset is a comprehensive collection of over 28,412 ear images from 164 individuals. It encompasses a wide range of variations in pose, scale, illumination, occlusion, resolution, and lighting conditions. This dataset is suitable for various applications such as person authentication and classification. This file contains a sample subset of EarVN1.0 that consists of selected ear images from male participants (sampled from Person IDs 1–98). The images capture a variety of angles, lighting conditions, and backgrounds to ensure diversity and support robust model training for male ear recognition tasks. [file peerj-cs-10-2603-s002.zip › 075.Rym/075 (13).jpg]

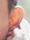

Supplement: Supplemental Information 2 — The EarVN1.0 dataset is a comprehensive collection of over 28,412 ear images from 164 individuals. It encompasses a wide range of variations in pose, scale, illumination, occlusion, resolution, and lighting conditions. This dataset is suitable for various applications such as person authentication and classification. This file contains a sample subset of EarVN1.0 that consists of selected ear images from male participants (sampled from Person IDs 1–98). The images capture a variety of angles, lighting conditions, and backgrounds to ensure diversity and support robust model training for male ear recognition tasks. [file peerj-cs-10-2603-s002.zip › 075.Rym/075 (130).jpg]

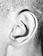

Supplement: Supplemental Information 2 — The EarVN1.0 dataset is a comprehensive collection of over 28,412 ear images from 164 individuals. It encompasses a wide range of variations in pose, scale, illumination, occlusion, resolution, and lighting conditions. This dataset is suitable for various applications such as person authentication and classification. This file contains a sample subset of EarVN1.0 that consists of selected ear images from male participants (sampled from Person IDs 1–98). The images capture a variety of angles, lighting conditions, and backgrounds to ensure diversity and support robust model training for male ear recognition tasks. [file peerj-cs-10-2603-s002.zip › 075.Rym/075 (131).jpg]

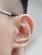

Supplement: Supplemental Information 2 — The EarVN1.0 dataset is a comprehensive collection of over 28,412 ear images from 164 individuals. It encompasses a wide range of variations in pose, scale, illumination, occlusion, resolution, and lighting conditions. This dataset is suitable for various applications such as person authentication and classification. This file contains a sample subset of EarVN1.0 that consists of selected ear images from male participants (sampled from Person IDs 1–98). The images capture a variety of angles, lighting conditions, and backgrounds to ensure diversity and support robust model training for male ear recognition tasks. [file peerj-cs-10-2603-s002.zip › 075.Rym/075 (132).jpg]

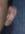

Supplement: Supplemental Information 2 — The EarVN1.0 dataset is a comprehensive collection of over 28,412 ear images from 164 individuals. It encompasses a wide range of variations in pose, scale, illumination, occlusion, resolution, and lighting conditions. This dataset is suitable for various applications such as person authentication and classification. This file contains a sample subset of EarVN1.0 that consists of selected ear images from male participants (sampled from Person IDs 1–98). The images capture a variety of angles, lighting conditions, and backgrounds to ensure diversity and support robust model training for male ear recognition tasks. [file peerj-cs-10-2603-s002.zip › 075.Rym/075 (133).jpg]

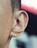

Supplement: Supplemental Information 2 — The EarVN1.0 dataset is a comprehensive collection of over 28,412 ear images from 164 individuals. It encompasses a wide range of variations in pose, scale, illumination, occlusion, resolution, and lighting conditions. This dataset is suitable for various applications such as person authentication and classification. This file contains a sample subset of EarVN1.0 that consists of selected ear images from male participants (sampled from Person IDs 1–98). The images capture a variety of angles, lighting conditions, and backgrounds to ensure diversity and support robust model training for male ear recognition tasks. [file peerj-cs-10-2603-s002.zip › 075.Rym/075 (134).jpg]

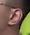

Supplement: Supplemental Information 2 — The EarVN1.0 dataset is a comprehensive collection of over 28,412 ear images from 164 individuals. It encompasses a wide range of variations in pose, scale, illumination, occlusion, resolution, and lighting conditions. This dataset is suitable for various applications such as person authentication and classification. This file contains a sample subset of EarVN1.0 that consists of selected ear images from male participants (sampled from Person IDs 1–98). The images capture a variety of angles, lighting conditions, and backgrounds to ensure diversity and support robust model training for male ear recognition tasks. [file peerj-cs-10-2603-s002.zip › 075.Rym/075 (135).jpg]

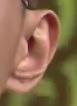

Supplement: Supplemental Information 2 — The EarVN1.0 dataset is a comprehensive collection of over 28,412 ear images from 164 individuals. It encompasses a wide range of variations in pose, scale, illumination, occlusion, resolution, and lighting conditions. This dataset is suitable for various applications such as person authentication and classification. This file contains a sample subset of EarVN1.0 that consists of selected ear images from male participants (sampled from Person IDs 1–98). The images capture a variety of angles, lighting conditions, and backgrounds to ensure diversity and support robust model training for male ear recognition tasks. [file peerj-cs-10-2603-s002.zip › 075.Rym/075 (136).jpg]

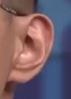

Supplement: Supplemental Information 2 — The EarVN1.0 dataset is a comprehensive collection of over 28,412 ear images from 164 individuals. It encompasses a wide range of variations in pose, scale, illumination, occlusion, resolution, and lighting conditions. This dataset is suitable for various applications such as person authentication and classification. This file contains a sample subset of EarVN1.0 that consists of selected ear images from male participants (sampled from Person IDs 1–98). The images capture a variety of angles, lighting conditions, and backgrounds to ensure diversity and support robust model training for male ear recognition tasks. [file peerj-cs-10-2603-s002.zip › 075.Rym/075 (137).jpg]

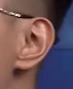

Supplement: Supplemental Information 2 — The EarVN1.0 dataset is a comprehensive collection of over 28,412 ear images from 164 individuals. It encompasses a wide range of variations in pose, scale, illumination, occlusion, resolution, and lighting conditions. This dataset is suitable for various applications such as person authentication and classification. This file contains a sample subset of EarVN1.0 that consists of selected ear images from male participants (sampled from Person IDs 1–98). The images capture a variety of angles, lighting conditions, and backgrounds to ensure diversity and support robust model training for male ear recognition tasks. [file peerj-cs-10-2603-s002.zip › 075.Rym/075 (138).jpg]

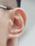

Supplement: Supplemental Information 2 — The EarVN1.0 dataset is a comprehensive collection of over 28,412 ear images from 164 individuals. It encompasses a wide range of variations in pose, scale, illumination, occlusion, resolution, and lighting conditions. This dataset is suitable for various applications such as person authentication and classification. This file contains a sample subset of EarVN1.0 that consists of selected ear images from male participants (sampled from Person IDs 1–98). The images capture a variety of angles, lighting conditions, and backgrounds to ensure diversity and support robust model training for male ear recognition tasks. [file peerj-cs-10-2603-s002.zip › 075.Rym/075 (139).jpg]

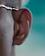

Supplement: Supplemental Information 2 — The EarVN1.0 dataset is a comprehensive collection of over 28,412 ear images from 164 individuals. It encompasses a wide range of variations in pose, scale, illumination, occlusion, resolution, and lighting conditions. This dataset is suitable for various applications such as person authentication and classification. This file contains a sample subset of EarVN1.0 that consists of selected ear images from male participants (sampled from Person IDs 1–98). The images capture a variety of angles, lighting conditions, and backgrounds to ensure diversity and support robust model training for male ear recognition tasks. [file peerj-cs-10-2603-s002.zip › 075.Rym/075 (14).jpg]

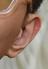

Supplement: Supplemental Information 2 — The EarVN1.0 dataset is a comprehensive collection of over 28,412 ear images from 164 individuals. It encompasses a wide range of variations in pose, scale, illumination, occlusion, resolution, and lighting conditions. This dataset is suitable for various applications such as person authentication and classification. This file contains a sample subset of EarVN1.0 that consists of selected ear images from male participants (sampled from Person IDs 1–98). The images capture a variety of angles, lighting conditions, and backgrounds to ensure diversity and support robust model training for male ear recognition tasks. [file peerj-cs-10-2603-s002.zip › 075.Rym/075 (140).jpg]

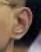

Supplement: Supplemental Information 2 — The EarVN1.0 dataset is a comprehensive collection of over 28,412 ear images from 164 individuals. It encompasses a wide range of variations in pose, scale, illumination, occlusion, resolution, and lighting conditions. This dataset is suitable for various applications such as person authentication and classification. This file contains a sample subset of EarVN1.0 that consists of selected ear images from male participants (sampled from Person IDs 1–98). The images capture a variety of angles, lighting conditions, and backgrounds to ensure diversity and support robust model training for male ear recognition tasks. [file peerj-cs-10-2603-s002.zip › 075.Rym/075 (141).jpg]

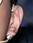

Supplement: Supplemental Information 2 — The EarVN1.0 dataset is a comprehensive collection of over 28,412 ear images from 164 individuals. It encompasses a wide range of variations in pose, scale, illumination, occlusion, resolution, and lighting conditions. This dataset is suitable for various applications such as person authentication and classification. This file contains a sample subset of EarVN1.0 that consists of selected ear images from male participants (sampled from Person IDs 1–98). The images capture a variety of angles, lighting conditions, and backgrounds to ensure diversity and support robust model training for male ear recognition tasks. [file peerj-cs-10-2603-s002.zip › 075.Rym/075 (142).jpg]

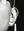

Supplement: Supplemental Information 2 — The EarVN1.0 dataset is a comprehensive collection of over 28,412 ear images from 164 individuals. It encompasses a wide range of variations in pose, scale, illumination, occlusion, resolution, and lighting conditions. This dataset is suitable for various applications such as person authentication and classification. This file contains a sample subset of EarVN1.0 that consists of selected ear images from male participants (sampled from Person IDs 1–98). The images capture a variety of angles, lighting conditions, and backgrounds to ensure diversity and support robust model training for male ear recognition tasks. [file peerj-cs-10-2603-s002.zip › 075.Rym/075 (143).jpg]

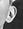

Supplement: Supplemental Information 2 — The EarVN1.0 dataset is a comprehensive collection of over 28,412 ear images from 164 individuals. It encompasses a wide range of variations in pose, scale, illumination, occlusion, resolution, and lighting conditions. This dataset is suitable for various applications such as person authentication and classification. This file contains a sample subset of EarVN1.0 that consists of selected ear images from male participants (sampled from Person IDs 1–98). The images capture a variety of angles, lighting conditions, and backgrounds to ensure diversity and support robust model training for male ear recognition tasks. [file peerj-cs-10-2603-s002.zip › 075.Rym/075 (144).jpg]

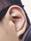

Supplement: Supplemental Information 2 — The EarVN1.0 dataset is a comprehensive collection of over 28,412 ear images from 164 individuals. It encompasses a wide range of variations in pose, scale, illumination, occlusion, resolution, and lighting conditions. This dataset is suitable for various applications such as person authentication and classification. This file contains a sample subset of EarVN1.0 that consists of selected ear images from male participants (sampled from Person IDs 1–98). The images capture a variety of angles, lighting conditions, and backgrounds to ensure diversity and support robust model training for male ear recognition tasks. [file peerj-cs-10-2603-s002.zip › 075.Rym/075 (145).jpg]

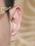

Supplement: Supplemental Information 2 — The EarVN1.0 dataset is a comprehensive collection of over 28,412 ear images from 164 individuals. It encompasses a wide range of variations in pose, scale, illumination, occlusion, resolution, and lighting conditions. This dataset is suitable for various applications such as person authentication and classification. This file contains a sample subset of EarVN1.0 that consists of selected ear images from male participants (sampled from Person IDs 1–98). The images capture a variety of angles, lighting conditions, and backgrounds to ensure diversity and support robust model training for male ear recognition tasks. [file peerj-cs-10-2603-s002.zip › 075.Rym/075 (146).jpg]

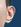

Supplement: Supplemental Information 2 — The EarVN1.0 dataset is a comprehensive collection of over 28,412 ear images from 164 individuals. It encompasses a wide range of variations in pose, scale, illumination, occlusion, resolution, and lighting conditions. This dataset is suitable for various applications such as person authentication and classification. This file contains a sample subset of EarVN1.0 that consists of selected ear images from male participants (sampled from Person IDs 1–98). The images capture a variety of angles, lighting conditions, and backgrounds to ensure diversity and support robust model training for male ear recognition tasks. [file peerj-cs-10-2603-s002.zip › 075.Rym/075 (147).jpg]

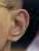

Supplement: Supplemental Information 2 — The EarVN1.0 dataset is a comprehensive collection of over 28,412 ear images from 164 individuals. It encompasses a wide range of variations in pose, scale, illumination, occlusion, resolution, and lighting conditions. This dataset is suitable for various applications such as person authentication and classification. This file contains a sample subset of EarVN1.0 that consists of selected ear images from male participants (sampled from Person IDs 1–98). The images capture a variety of angles, lighting conditions, and backgrounds to ensure diversity and support robust model training for male ear recognition tasks. [file peerj-cs-10-2603-s002.zip › 075.Rym/075 (148).jpg]

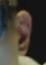

Supplement: Supplemental Information 2 — The EarVN1.0 dataset is a comprehensive collection of over 28,412 ear images from 164 individuals. It encompasses a wide range of variations in pose, scale, illumination, occlusion, resolution, and lighting conditions. This dataset is suitable for various applications such as person authentication and classification. This file contains a sample subset of EarVN1.0 that consists of selected ear images from male participants (sampled from Person IDs 1–98). The images capture a variety of angles, lighting conditions, and backgrounds to ensure diversity and support robust model training for male ear recognition tasks. [file peerj-cs-10-2603-s002.zip › 075.Rym/075 (149).jpg]

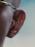

Supplement: Supplemental Information 2 — The EarVN1.0 dataset is a comprehensive collection of over 28,412 ear images from 164 individuals. It encompasses a wide range of variations in pose, scale, illumination, occlusion, resolution, and lighting conditions. This dataset is suitable for various applications such as person authentication and classification. This file contains a sample subset of EarVN1.0 that consists of selected ear images from male participants (sampled from Person IDs 1–98). The images capture a variety of angles, lighting conditions, and backgrounds to ensure diversity and support robust model training for male ear recognition tasks. [file peerj-cs-10-2603-s002.zip › 075.Rym/075 (15).jpg]

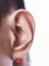

Supplement: Supplemental Information 2 — The EarVN1.0 dataset is a comprehensive collection of over 28,412 ear images from 164 individuals. It encompasses a wide range of variations in pose, scale, illumination, occlusion, resolution, and lighting conditions. This dataset is suitable for various applications such as person authentication and classification. This file contains a sample subset of EarVN1.0 that consists of selected ear images from male participants (sampled from Person IDs 1–98). The images capture a variety of angles, lighting conditions, and backgrounds to ensure diversity and support robust model training for male ear recognition tasks. [file peerj-cs-10-2603-s002.zip › 075.Rym/075 (150).jpg]

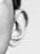

Supplement: Supplemental Information 2 — The EarVN1.0 dataset is a comprehensive collection of over 28,412 ear images from 164 individuals. It encompasses a wide range of variations in pose, scale, illumination, occlusion, resolution, and lighting conditions. This dataset is suitable for various applications such as person authentication and classification. This file contains a sample subset of EarVN1.0 that consists of selected ear images from male participants (sampled from Person IDs 1–98). The images capture a variety of angles, lighting conditions, and backgrounds to ensure diversity and support robust model training for male ear recognition tasks. [file peerj-cs-10-2603-s002.zip › 075.Rym/075 (151).jpg]

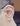

Supplement: Supplemental Information 2 — The EarVN1.0 dataset is a comprehensive collection of over 28,412 ear images from 164 individuals. It encompasses a wide range of variations in pose, scale, illumination, occlusion, resolution, and lighting conditions. This dataset is suitable for various applications such as person authentication and classification. This file contains a sample subset of EarVN1.0 that consists of selected ear images from male participants (sampled from Person IDs 1–98). The images capture a variety of angles, lighting conditions, and backgrounds to ensure diversity and support robust model training for male ear recognition tasks. [file peerj-cs-10-2603-s002.zip › 075.Rym/075 (152).jpg]

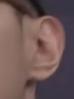

Supplement: Supplemental Information 2 — The EarVN1.0 dataset is a comprehensive collection of over 28,412 ear images from 164 individuals. It encompasses a wide range of variations in pose, scale, illumination, occlusion, resolution, and lighting conditions. This dataset is suitable for various applications such as person authentication and classification. This file contains a sample subset of EarVN1.0 that consists of selected ear images from male participants (sampled from Person IDs 1–98). The images capture a variety of angles, lighting conditions, and backgrounds to ensure diversity and support robust model training for male ear recognition tasks. [file peerj-cs-10-2603-s002.zip › 075.Rym/075 (153).jpg]

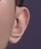

Supplement: Supplemental Information 2 — The EarVN1.0 dataset is a comprehensive collection of over 28,412 ear images from 164 individuals. It encompasses a wide range of variations in pose, scale, illumination, occlusion, resolution, and lighting conditions. This dataset is suitable for various applications such as person authentication and classification. This file contains a sample subset of EarVN1.0 that consists of selected ear images from male participants (sampled from Person IDs 1–98). The images capture a variety of angles, lighting conditions, and backgrounds to ensure diversity and support robust model training for male ear recognition tasks. [file peerj-cs-10-2603-s002.zip › 075.Rym/075 (154).jpg]

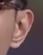

Supplement: Supplemental Information 2 — The EarVN1.0 dataset is a comprehensive collection of over 28,412 ear images from 164 individuals. It encompasses a wide range of variations in pose, scale, illumination, occlusion, resolution, and lighting conditions. This dataset is suitable for various applications such as person authentication and classification. This file contains a sample subset of EarVN1.0 that consists of selected ear images from male participants (sampled from Person IDs 1–98). The images capture a variety of angles, lighting conditions, and backgrounds to ensure diversity and support robust model training for male ear recognition tasks. [file peerj-cs-10-2603-s002.zip › 075.Rym/075 (155).jpg]

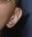

Supplement: Supplemental Information 2 — The EarVN1.0 dataset is a comprehensive collection of over 28,412 ear images from 164 individuals. It encompasses a wide range of variations in pose, scale, illumination, occlusion, resolution, and lighting conditions. This dataset is suitable for various applications such as person authentication and classification. This file contains a sample subset of EarVN1.0 that consists of selected ear images from male participants (sampled from Person IDs 1–98). The images capture a variety of angles, lighting conditions, and backgrounds to ensure diversity and support robust model training for male ear recognition tasks. [file peerj-cs-10-2603-s002.zip › 075.Rym/075 (156).jpg]

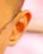

Supplement: Supplemental Information 2 — The EarVN1.0 dataset is a comprehensive collection of over 28,412 ear images from 164 individuals. It encompasses a wide range of variations in pose, scale, illumination, occlusion, resolution, and lighting conditions. This dataset is suitable for various applications such as person authentication and classification. This file contains a sample subset of EarVN1.0 that consists of selected ear images from male participants (sampled from Person IDs 1–98). The images capture a variety of angles, lighting conditions, and backgrounds to ensure diversity and support robust model training for male ear recognition tasks. [file peerj-cs-10-2603-s002.zip › 075.Rym/075 (157).jpg]

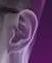

Supplement: Supplemental Information 2 — The EarVN1.0 dataset is a comprehensive collection of over 28,412 ear images from 164 individuals. It encompasses a wide range of variations in pose, scale, illumination, occlusion, resolution, and lighting conditions. This dataset is suitable for various applications such as person authentication and classification. This file contains a sample subset of EarVN1.0 that consists of selected ear images from male participants (sampled from Person IDs 1–98). The images capture a variety of angles, lighting conditions, and backgrounds to ensure diversity and support robust model training for male ear recognition tasks. [file peerj-cs-10-2603-s002.zip › 075.Rym/075 (158).jpg]

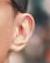

Supplement: Supplemental Information 2 — The EarVN1.0 dataset is a comprehensive collection of over 28,412 ear images from 164 individuals. It encompasses a wide range of variations in pose, scale, illumination, occlusion, resolution, and lighting conditions. This dataset is suitable for various applications such as person authentication and classification. This file contains a sample subset of EarVN1.0 that consists of selected ear images from male participants (sampled from Person IDs 1–98). The images capture a variety of angles, lighting conditions, and backgrounds to ensure diversity and support robust model training for male ear recognition tasks. [file peerj-cs-10-2603-s002.zip › 075.Rym/075 (159).jpg]

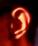

Supplement: Supplemental Information 2 — The EarVN1.0 dataset is a comprehensive collection of over 28,412 ear images from 164 individuals. It encompasses a wide range of variations in pose, scale, illumination, occlusion, resolution, and lighting conditions. This dataset is suitable for various applications such as person authentication and classification. This file contains a sample subset of EarVN1.0 that consists of selected ear images from male participants (sampled from Person IDs 1–98). The images capture a variety of angles, lighting conditions, and backgrounds to ensure diversity and support robust model training for male ear recognition tasks. [file peerj-cs-10-2603-s002.zip › 075.Rym/075 (16).jpg]

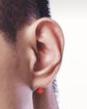

Supplement: Supplemental Information 2 — The EarVN1.0 dataset is a comprehensive collection of over 28,412 ear images from 164 individuals. It encompasses a wide range of variations in pose, scale, illumination, occlusion, resolution, and lighting conditions. This dataset is suitable for various applications such as person authentication and classification. This file contains a sample subset of EarVN1.0 that consists of selected ear images from male participants (sampled from Person IDs 1–98). The images capture a variety of angles, lighting conditions, and backgrounds to ensure diversity and support robust model training for male ear recognition tasks. [file peerj-cs-10-2603-s002.zip › 075.Rym/075 (160).jpg]

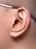

Supplement: Supplemental Information 2 — The EarVN1.0 dataset is a comprehensive collection of over 28,412 ear images from 164 individuals. It encompasses a wide range of variations in pose, scale, illumination, occlusion, resolution, and lighting conditions. This dataset is suitable for various applications such as person authentication and classification. This file contains a sample subset of EarVN1.0 that consists of selected ear images from male participants (sampled from Person IDs 1–98). The images capture a variety of angles, lighting conditions, and backgrounds to ensure diversity and support robust model training for male ear recognition tasks. [file peerj-cs-10-2603-s002.zip › 075.Rym/075 (17).jpg]

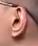

Supplement: Supplemental Information 2 — The EarVN1.0 dataset is a comprehensive collection of over 28,412 ear images from 164 individuals. It encompasses a wide range of variations in pose, scale, illumination, occlusion, resolution, and lighting conditions. This dataset is suitable for various applications such as person authentication and classification. This file contains a sample subset of EarVN1.0 that consists of selected ear images from male participants (sampled from Person IDs 1–98). The images capture a variety of angles, lighting conditions, and backgrounds to ensure diversity and support robust model training for male ear recognition tasks. [file peerj-cs-10-2603-s002.zip › 075.Rym/075 (18).jpg]

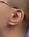

Supplement: Supplemental Information 2 — The EarVN1.0 dataset is a comprehensive collection of over 28,412 ear images from 164 individuals. It encompasses a wide range of variations in pose, scale, illumination, occlusion, resolution, and lighting conditions. This dataset is suitable for various applications such as person authentication and classification. This file contains a sample subset of EarVN1.0 that consists of selected ear images from male participants (sampled from Person IDs 1–98). The images capture a variety of angles, lighting conditions, and backgrounds to ensure diversity and support robust model training for male ear recognition tasks. [file peerj-cs-10-2603-s002.zip › 075.Rym/075 (19).jpg]

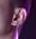

Supplement: Supplemental Information 2 — The EarVN1.0 dataset is a comprehensive collection of over 28,412 ear images from 164 individuals. It encompasses a wide range of variations in pose, scale, illumination, occlusion, resolution, and lighting conditions. This dataset is suitable for various applications such as person authentication and classification. This file contains a sample subset of EarVN1.0 that consists of selected ear images from male participants (sampled from Person IDs 1–98). The images capture a variety of angles, lighting conditions, and backgrounds to ensure diversity and support robust model training for male ear recognition tasks. [file peerj-cs-10-2603-s002.zip › 075.Rym/075 (2).jpg]

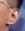

Supplement: Supplemental Information 2 — The EarVN1.0 dataset is a comprehensive collection of over 28,412 ear images from 164 individuals. It encompasses a wide range of variations in pose, scale, illumination, occlusion, resolution, and lighting conditions. This dataset is suitable for various applications such as person authentication and classification. This file contains a sample subset of EarVN1.0 that consists of selected ear images from male participants (sampled from Person IDs 1–98). The images capture a variety of angles, lighting conditions, and backgrounds to ensure diversity and support robust model training for male ear recognition tasks. [file peerj-cs-10-2603-s002.zip › 075.Rym/075 (20).jpg]

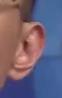

Supplement: Supplemental Information 2 — The EarVN1.0 dataset is a comprehensive collection of over 28,412 ear images from 164 individuals. It encompasses a wide range of variations in pose, scale, illumination, occlusion, resolution, and lighting conditions. This dataset is suitable for various applications such as person authentication and classification. This file contains a sample subset of EarVN1.0 that consists of selected ear images from male participants (sampled from Person IDs 1–98). The images capture a variety of angles, lighting conditions, and backgrounds to ensure diversity and support robust model training for male ear recognition tasks. [file peerj-cs-10-2603-s002.zip › 075.Rym/075 (21).jpg]

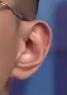

Supplement: Supplemental Information 2 — The EarVN1.0 dataset is a comprehensive collection of over 28,412 ear images from 164 individuals. It encompasses a wide range of variations in pose, scale, illumination, occlusion, resolution, and lighting conditions. This dataset is suitable for various applications such as person authentication and classification. This file contains a sample subset of EarVN1.0 that consists of selected ear images from male participants (sampled from Person IDs 1–98). The images capture a variety of angles, lighting conditions, and backgrounds to ensure diversity and support robust model training for male ear recognition tasks. [file peerj-cs-10-2603-s002.zip › 075.Rym/075 (22).jpg]

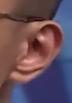

Supplement: Supplemental Information 2 — The EarVN1.0 dataset is a comprehensive collection of over 28,412 ear images from 164 individuals. It encompasses a wide range of variations in pose, scale, illumination, occlusion, resolution, and lighting conditions. This dataset is suitable for various applications such as person authentication and classification. This file contains a sample subset of EarVN1.0 that consists of selected ear images from male participants (sampled from Person IDs 1–98). The images capture a variety of angles, lighting conditions, and backgrounds to ensure diversity and support robust model training for male ear recognition tasks. [file peerj-cs-10-2603-s002.zip › 075.Rym/075 (23).jpg]

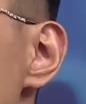

Supplement: Supplemental Information 2 — The EarVN1.0 dataset is a comprehensive collection of over 28,412 ear images from 164 individuals. It encompasses a wide range of variations in pose, scale, illumination, occlusion, resolution, and lighting conditions. This dataset is suitable for various applications such as person authentication and classification. This file contains a sample subset of EarVN1.0 that consists of selected ear images from male participants (sampled from Person IDs 1–98). The images capture a variety of angles, lighting conditions, and backgrounds to ensure diversity and support robust model training for male ear recognition tasks. [file peerj-cs-10-2603-s002.zip › 075.Rym/075 (24).jpg]

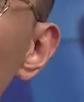

Supplement: Supplemental Information 2 — The EarVN1.0 dataset is a comprehensive collection of over 28,412 ear images from 164 individuals. It encompasses a wide range of variations in pose, scale, illumination, occlusion, resolution, and lighting conditions. This dataset is suitable for various applications such as person authentication and classification. This file contains a sample subset of EarVN1.0 that consists of selected ear images from male participants (sampled from Person IDs 1–98). The images capture a variety of angles, lighting conditions, and backgrounds to ensure diversity and support robust model training for male ear recognition tasks. [file peerj-cs-10-2603-s002.zip › 075.Rym/075 (25).jpg]

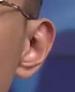

Supplement: Supplemental Information 2 — The EarVN1.0 dataset is a comprehensive collection of over 28,412 ear images from 164 individuals. It encompasses a wide range of variations in pose, scale, illumination, occlusion, resolution, and lighting conditions. This dataset is suitable for various applications such as person authentication and classification. This file contains a sample subset of EarVN1.0 that consists of selected ear images from male participants (sampled from Person IDs 1–98). The images capture a variety of angles, lighting conditions, and backgrounds to ensure diversity and support robust model training for male ear recognition tasks. [file peerj-cs-10-2603-s002.zip › 075.Rym/075 (26).jpg]

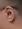

Supplement: Supplemental Information 2 — The EarVN1.0 dataset is a comprehensive collection of over 28,412 ear images from 164 individuals. It encompasses a wide range of variations in pose, scale, illumination, occlusion, resolution, and lighting conditions. This dataset is suitable for various applications such as person authentication and classification. This file contains a sample subset of EarVN1.0 that consists of selected ear images from male participants (sampled from Person IDs 1–98). The images capture a variety of angles, lighting conditions, and backgrounds to ensure diversity and support robust model training for male ear recognition tasks. [file peerj-cs-10-2603-s002.zip › 075.Rym/075 (27).jpg]

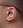

Supplement: Supplemental Information 2 — The EarVN1.0 dataset is a comprehensive collection of over 28,412 ear images from 164 individuals. It encompasses a wide range of variations in pose, scale, illumination, occlusion, resolution, and lighting conditions. This dataset is suitable for various applications such as person authentication and classification. This file contains a sample subset of EarVN1.0 that consists of selected ear images from male participants (sampled from Person IDs 1–98). The images capture a variety of angles, lighting conditions, and backgrounds to ensure diversity and support robust model training for male ear recognition tasks. [file peerj-cs-10-2603-s002.zip › 075.Rym/075 (28).jpg]

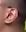

Supplement: Supplemental Information 2 — The EarVN1.0 dataset is a comprehensive collection of over 28,412 ear images from 164 individuals. It encompasses a wide range of variations in pose, scale, illumination, occlusion, resolution, and lighting conditions. This dataset is suitable for various applications such as person authentication and classification. This file contains a sample subset of EarVN1.0 that consists of selected ear images from male participants (sampled from Person IDs 1–98). The images capture a variety of angles, lighting conditions, and backgrounds to ensure diversity and support robust model training for male ear recognition tasks. [file peerj-cs-10-2603-s002.zip › 075.Rym/075 (29).jpg]

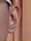

Supplement: Supplemental Information 2 — The EarVN1.0 dataset is a comprehensive collection of over 28,412 ear images from 164 individuals. It encompasses a wide range of variations in pose, scale, illumination, occlusion, resolution, and lighting conditions. This dataset is suitable for various applications such as person authentication and classification. This file contains a sample subset of EarVN1.0 that consists of selected ear images from male participants (sampled from Person IDs 1–98). The images capture a variety of angles, lighting conditions, and backgrounds to ensure diversity and support robust model training for male ear recognition tasks. [file peerj-cs-10-2603-s002.zip › 075.Rym/075 (3).jpg]

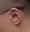

Supplement: Supplemental Information 2 — The EarVN1.0 dataset is a comprehensive collection of over 28,412 ear images from 164 individuals. It encompasses a wide range of variations in pose, scale, illumination, occlusion, resolution, and lighting conditions. This dataset is suitable for various applications such as person authentication and classification. This file contains a sample subset of EarVN1.0 that consists of selected ear images from male participants (sampled from Person IDs 1–98). The images capture a variety of angles, lighting conditions, and backgrounds to ensure diversity and support robust model training for male ear recognition tasks. [file peerj-cs-10-2603-s002.zip › 075.Rym/075 (30).jpg]

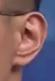

Supplement: Supplemental Information 2 — The EarVN1.0 dataset is a comprehensive collection of over 28,412 ear images from 164 individuals. It encompasses a wide range of variations in pose, scale, illumination, occlusion, resolution, and lighting conditions. This dataset is suitable for various applications such as person authentication and classification. This file contains a sample subset of EarVN1.0 that consists of selected ear images from male participants (sampled from Person IDs 1–98). The images capture a variety of angles, lighting conditions, and backgrounds to ensure diversity and support robust model training for male ear recognition tasks. [file peerj-cs-10-2603-s002.zip › 075.Rym/075 (31).jpg]

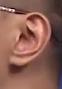

Supplement: Supplemental Information 2 — The EarVN1.0 dataset is a comprehensive collection of over 28,412 ear images from 164 individuals. It encompasses a wide range of variations in pose, scale, illumination, occlusion, resolution, and lighting conditions. This dataset is suitable for various applications such as person authentication and classification. This file contains a sample subset of EarVN1.0 that consists of selected ear images from male participants (sampled from Person IDs 1–98). The images capture a variety of angles, lighting conditions, and backgrounds to ensure diversity and support robust model training for male ear recognition tasks. [file peerj-cs-10-2603-s002.zip › 075.Rym/075 (32).jpg]

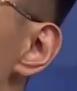

Supplement: Supplemental Information 2 — The EarVN1.0 dataset is a comprehensive collection of over 28,412 ear images from 164 individuals. It encompasses a wide range of variations in pose, scale, illumination, occlusion, resolution, and lighting conditions. This dataset is suitable for various applications such as person authentication and classification. This file contains a sample subset of EarVN1.0 that consists of selected ear images from male participants (sampled from Person IDs 1–98). The images capture a variety of angles, lighting conditions, and backgrounds to ensure diversity and support robust model training for male ear recognition tasks. [file peerj-cs-10-2603-s002.zip › 075.Rym/075 (33).jpg]

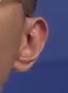

Supplement: Supplemental Information 2 — The EarVN1.0 dataset is a comprehensive collection of over 28,412 ear images from 164 individuals. It encompasses a wide range of variations in pose, scale, illumination, occlusion, resolution, and lighting conditions. This dataset is suitable for various applications such as person authentication and classification. This file contains a sample subset of EarVN1.0 that consists of selected ear images from male participants (sampled from Person IDs 1–98). The images capture a variety of angles, lighting conditions, and backgrounds to ensure diversity and support robust model training for male ear recognition tasks. [file peerj-cs-10-2603-s002.zip › 075.Rym/075 (34).jpg]

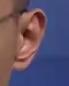

Supplement: Supplemental Information 2 — The EarVN1.0 dataset is a comprehensive collection of over 28,412 ear images from 164 individuals. It encompasses a wide range of variations in pose, scale, illumination, occlusion, resolution, and lighting conditions. This dataset is suitable for various applications such as person authentication and classification. This file contains a sample subset of EarVN1.0 that consists of selected ear images from male participants (sampled from Person IDs 1–98). The images capture a variety of angles, lighting conditions, and backgrounds to ensure diversity and support robust model training for male ear recognition tasks. [file peerj-cs-10-2603-s002.zip › 075.Rym/075 (35).jpg]

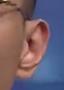

Supplement: Supplemental Information 2 — The EarVN1.0 dataset is a comprehensive collection of over 28,412 ear images from 164 individuals. It encompasses a wide range of variations in pose, scale, illumination, occlusion, resolution, and lighting conditions. This dataset is suitable for various applications such as person authentication and classification. This file contains a sample subset of EarVN1.0 that consists of selected ear images from male participants (sampled from Person IDs 1–98). The images capture a variety of angles, lighting conditions, and backgrounds to ensure diversity and support robust model training for male ear recognition tasks. [file peerj-cs-10-2603-s002.zip › 075.Rym/075 (36).jpg]

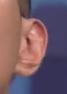

Supplement: Supplemental Information 2 — The EarVN1.0 dataset is a comprehensive collection of over 28,412 ear images from 164 individuals. It encompasses a wide range of variations in pose, scale, illumination, occlusion, resolution, and lighting conditions. This dataset is suitable for various applications such as person authentication and classification. This file contains a sample subset of EarVN1.0 that consists of selected ear images from male participants (sampled from Person IDs 1–98). The images capture a variety of angles, lighting conditions, and backgrounds to ensure diversity and support robust model training for male ear recognition tasks. [file peerj-cs-10-2603-s002.zip › 075.Rym/075 (37).jpg]

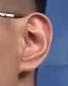

Supplement: Supplemental Information 2 — The EarVN1.0 dataset is a comprehensive collection of over 28,412 ear images from 164 individuals. It encompasses a wide range of variations in pose, scale, illumination, occlusion, resolution, and lighting conditions. This dataset is suitable for various applications such as person authentication and classification. This file contains a sample subset of EarVN1.0 that consists of selected ear images from male participants (sampled from Person IDs 1–98). The images capture a variety of angles, lighting conditions, and backgrounds to ensure diversity and support robust model training for male ear recognition tasks. [file peerj-cs-10-2603-s002.zip › 075.Rym/075 (38).jpg]

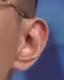

Supplement: Supplemental Information 2 — The EarVN1.0 dataset is a comprehensive collection of over 28,412 ear images from 164 individuals. It encompasses a wide range of variations in pose, scale, illumination, occlusion, resolution, and lighting conditions. This dataset is suitable for various applications such as person authentication and classification. This file contains a sample subset of EarVN1.0 that consists of selected ear images from male participants (sampled from Person IDs 1–98). The images capture a variety of angles, lighting conditions, and backgrounds to ensure diversity and support robust model training for male ear recognition tasks. [file peerj-cs-10-2603-s002.zip › 075.Rym/075 (39).jpg]

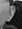

Supplement: Supplemental Information 2 — The EarVN1.0 dataset is a comprehensive collection of over 28,412 ear images from 164 individuals. It encompasses a wide range of variations in pose, scale, illumination, occlusion, resolution, and lighting conditions. This dataset is suitable for various applications such as person authentication and classification. This file contains a sample subset of EarVN1.0 that consists of selected ear images from male participants (sampled from Person IDs 1–98). The images capture a variety of angles, lighting conditions, and backgrounds to ensure diversity and support robust model training for male ear recognition tasks. [file peerj-cs-10-2603-s002.zip › 075.Rym/075 (4).jpg]

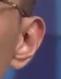

Supplement: Supplemental Information 2 — The EarVN1.0 dataset is a comprehensive collection of over 28,412 ear images from 164 individuals. It encompasses a wide range of variations in pose, scale, illumination, occlusion, resolution, and lighting conditions. This dataset is suitable for various applications such as person authentication and classification. This file contains a sample subset of EarVN1.0 that consists of selected ear images from male participants (sampled from Person IDs 1–98). The images capture a variety of angles, lighting conditions, and backgrounds to ensure diversity and support robust model training for male ear recognition tasks. [file peerj-cs-10-2603-s002.zip › 075.Rym/075 (40).jpg]

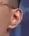

Supplement: Supplemental Information 2 — The EarVN1.0 dataset is a comprehensive collection of over 28,412 ear images from 164 individuals. It encompasses a wide range of variations in pose, scale, illumination, occlusion, resolution, and lighting conditions. This dataset is suitable for various applications such as person authentication and classification. This file contains a sample subset of EarVN1.0 that consists of selected ear images from male participants (sampled from Person IDs 1–98). The images capture a variety of angles, lighting conditions, and backgrounds to ensure diversity and support robust model training for male ear recognition tasks. [file peerj-cs-10-2603-s002.zip › 075.Rym/075 (41).jpg]

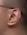

Supplement: Supplemental Information 2 — The EarVN1.0 dataset is a comprehensive collection of over 28,412 ear images from 164 individuals. It encompasses a wide range of variations in pose, scale, illumination, occlusion, resolution, and lighting conditions. This dataset is suitable for various applications such as person authentication and classification. This file contains a sample subset of EarVN1.0 that consists of selected ear images from male participants (sampled from Person IDs 1–98). The images capture a variety of angles, lighting conditions, and backgrounds to ensure diversity and support robust model training for male ear recognition tasks. [file peerj-cs-10-2603-s002.zip › 075.Rym/075 (42).jpg]

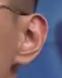

Supplement: Supplemental Information 2 — The EarVN1.0 dataset is a comprehensive collection of over 28,412 ear images from 164 individuals. It encompasses a wide range of variations in pose, scale, illumination, occlusion, resolution, and lighting conditions. This dataset is suitable for various applications such as person authentication and classification. This file contains a sample subset of EarVN1.0 that consists of selected ear images from male participants (sampled from Person IDs 1–98). The images capture a variety of angles, lighting conditions, and backgrounds to ensure diversity and support robust model training for male ear recognition tasks. [file peerj-cs-10-2603-s002.zip › 075.Rym/075 (43).jpg]

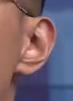

Supplement: Supplemental Information 2 — The EarVN1.0 dataset is a comprehensive collection of over 28,412 ear images from 164 individuals. It encompasses a wide range of variations in pose, scale, illumination, occlusion, resolution, and lighting conditions. This dataset is suitable for various applications such as person authentication and classification. This file contains a sample subset of EarVN1.0 that consists of selected ear images from male participants (sampled from Person IDs 1–98). The images capture a variety of angles, lighting conditions, and backgrounds to ensure diversity and support robust model training for male ear recognition tasks. [file peerj-cs-10-2603-s002.zip › 075.Rym/075 (44).jpg]
