# Supplementary figures and images for: DANNET: deep attention neural network for efficient ear identification in biometrics (part 3 of 4)
Source: PeerJ Comput Sci. 2024 Dec 18;10:e2603. doi: 10.7717/peerj-cs.2603 (PMC11784740; doi:10.7717/peerj-cs.2603)

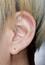

Supplement: Supplemental Information 3 — This file contains a sample subset of EarVN1.0 that includes selected ear images from female participants (sampled from Person IDs 99–164). The images are chosen to represent variations in pose, illumination, and environmental settings, offering a comprehensive foundation for female ear recognition studies. [file peerj-cs-10-2603-s003.zip › 099.Amber/099 (1).jpg]

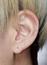

Supplement: Supplemental Information 3 — This file contains a sample subset of EarVN1.0 that includes selected ear images from female participants (sampled from Person IDs 99–164). The images are chosen to represent variations in pose, illumination, and environmental settings, offering a comprehensive foundation for female ear recognition studies. [file peerj-cs-10-2603-s003.zip › 099.Amber/099 (10).jpg]

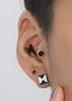

Supplement: Supplemental Information 3 — This file contains a sample subset of EarVN1.0 that includes selected ear images from female participants (sampled from Person IDs 99–164). The images are chosen to represent variations in pose, illumination, and environmental settings, offering a comprehensive foundation for female ear recognition studies. [file peerj-cs-10-2603-s003.zip › 099.Amber/099 (100).jpg]

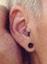

Supplement: Supplemental Information 3 — This file contains a sample subset of EarVN1.0 that includes selected ear images from female participants (sampled from Person IDs 99–164). The images are chosen to represent variations in pose, illumination, and environmental settings, offering a comprehensive foundation for female ear recognition studies. [file peerj-cs-10-2603-s003.zip › 099.Amber/099 (101).jpg]

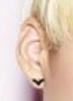

Supplement: Supplemental Information 3 — This file contains a sample subset of EarVN1.0 that includes selected ear images from female participants (sampled from Person IDs 99–164). The images are chosen to represent variations in pose, illumination, and environmental settings, offering a comprehensive foundation for female ear recognition studies. [file peerj-cs-10-2603-s003.zip › 099.Amber/099 (102).jpg]

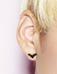

Supplement: Supplemental Information 3 — This file contains a sample subset of EarVN1.0 that includes selected ear images from female participants (sampled from Person IDs 99–164). The images are chosen to represent variations in pose, illumination, and environmental settings, offering a comprehensive foundation for female ear recognition studies. [file peerj-cs-10-2603-s003.zip › 099.Amber/099 (103).jpg]

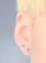

Supplement: Supplemental Information 3 — This file contains a sample subset of EarVN1.0 that includes selected ear images from female participants (sampled from Person IDs 99–164). The images are chosen to represent variations in pose, illumination, and environmental settings, offering a comprehensive foundation for female ear recognition studies. [file peerj-cs-10-2603-s003.zip › 099.Amber/099 (104).jpg]

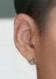

Supplement: Supplemental Information 3 — This file contains a sample subset of EarVN1.0 that includes selected ear images from female participants (sampled from Person IDs 99–164). The images are chosen to represent variations in pose, illumination, and environmental settings, offering a comprehensive foundation for female ear recognition studies. [file peerj-cs-10-2603-s003.zip › 099.Amber/099 (105).jpg]

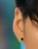

Supplement: Supplemental Information 3 — This file contains a sample subset of EarVN1.0 that includes selected ear images from female participants (sampled from Person IDs 99–164). The images are chosen to represent variations in pose, illumination, and environmental settings, offering a comprehensive foundation for female ear recognition studies. [file peerj-cs-10-2603-s003.zip › 099.Amber/099 (106).jpg]

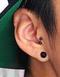

Supplement: Supplemental Information 3 — This file contains a sample subset of EarVN1.0 that includes selected ear images from female participants (sampled from Person IDs 99–164). The images are chosen to represent variations in pose, illumination, and environmental settings, offering a comprehensive foundation for female ear recognition studies. [file peerj-cs-10-2603-s003.zip › 099.Amber/099 (107).jpg]

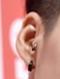

Supplement: Supplemental Information 3 — This file contains a sample subset of EarVN1.0 that includes selected ear images from female participants (sampled from Person IDs 99–164). The images are chosen to represent variations in pose, illumination, and environmental settings, offering a comprehensive foundation for female ear recognition studies. [file peerj-cs-10-2603-s003.zip › 099.Amber/099 (108).jpg]

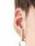

Supplement: Supplemental Information 3 — This file contains a sample subset of EarVN1.0 that includes selected ear images from female participants (sampled from Person IDs 99–164). The images are chosen to represent variations in pose, illumination, and environmental settings, offering a comprehensive foundation for female ear recognition studies. [file peerj-cs-10-2603-s003.zip › 099.Amber/099 (109).jpg]

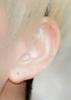

Supplement: Supplemental Information 3 — This file contains a sample subset of EarVN1.0 that includes selected ear images from female participants (sampled from Person IDs 99–164). The images are chosen to represent variations in pose, illumination, and environmental settings, offering a comprehensive foundation for female ear recognition studies. [file peerj-cs-10-2603-s003.zip › 099.Amber/099 (11).jpg]

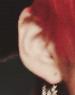

Supplement: Supplemental Information 3 — This file contains a sample subset of EarVN1.0 that includes selected ear images from female participants (sampled from Person IDs 99–164). The images are chosen to represent variations in pose, illumination, and environmental settings, offering a comprehensive foundation for female ear recognition studies. [file peerj-cs-10-2603-s003.zip › 099.Amber/099 (110).jpg]

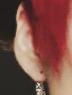

Supplement: Supplemental Information 3 — This file contains a sample subset of EarVN1.0 that includes selected ear images from female participants (sampled from Person IDs 99–164). The images are chosen to represent variations in pose, illumination, and environmental settings, offering a comprehensive foundation for female ear recognition studies. [file peerj-cs-10-2603-s003.zip › 099.Amber/099 (111).jpg]

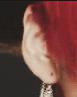

Supplement: Supplemental Information 3 — This file contains a sample subset of EarVN1.0 that includes selected ear images from female participants (sampled from Person IDs 99–164). The images are chosen to represent variations in pose, illumination, and environmental settings, offering a comprehensive foundation for female ear recognition studies. [file peerj-cs-10-2603-s003.zip › 099.Amber/099 (112).jpg]

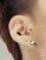

Supplement: Supplemental Information 3 — This file contains a sample subset of EarVN1.0 that includes selected ear images from female participants (sampled from Person IDs 99–164). The images are chosen to represent variations in pose, illumination, and environmental settings, offering a comprehensive foundation for female ear recognition studies. [file peerj-cs-10-2603-s003.zip › 099.Amber/099 (113).jpg]

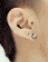

Supplement: Supplemental Information 3 — This file contains a sample subset of EarVN1.0 that includes selected ear images from female participants (sampled from Person IDs 99–164). The images are chosen to represent variations in pose, illumination, and environmental settings, offering a comprehensive foundation for female ear recognition studies. [file peerj-cs-10-2603-s003.zip › 099.Amber/099 (114).jpg]

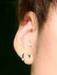

Supplement: Supplemental Information 3 — This file contains a sample subset of EarVN1.0 that includes selected ear images from female participants (sampled from Person IDs 99–164). The images are chosen to represent variations in pose, illumination, and environmental settings, offering a comprehensive foundation for female ear recognition studies. [file peerj-cs-10-2603-s003.zip › 099.Amber/099 (115).jpg]

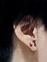

Supplement: Supplemental Information 3 — This file contains a sample subset of EarVN1.0 that includes selected ear images from female participants (sampled from Person IDs 99–164). The images are chosen to represent variations in pose, illumination, and environmental settings, offering a comprehensive foundation for female ear recognition studies. [file peerj-cs-10-2603-s003.zip › 099.Amber/099 (116).jpg]

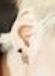

Supplement: Supplemental Information 3 — This file contains a sample subset of EarVN1.0 that includes selected ear images from female participants (sampled from Person IDs 99–164). The images are chosen to represent variations in pose, illumination, and environmental settings, offering a comprehensive foundation for female ear recognition studies. [file peerj-cs-10-2603-s003.zip › 099.Amber/099 (117).jpg]

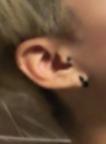

Supplement: Supplemental Information 3 — This file contains a sample subset of EarVN1.0 that includes selected ear images from female participants (sampled from Person IDs 99–164). The images are chosen to represent variations in pose, illumination, and environmental settings, offering a comprehensive foundation for female ear recognition studies. [file peerj-cs-10-2603-s003.zip › 099.Amber/099 (118).jpg]

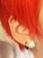

Supplement: Supplemental Information 3 — This file contains a sample subset of EarVN1.0 that includes selected ear images from female participants (sampled from Person IDs 99–164). The images are chosen to represent variations in pose, illumination, and environmental settings, offering a comprehensive foundation for female ear recognition studies. [file peerj-cs-10-2603-s003.zip › 099.Amber/099 (119).jpg]

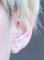

Supplement: Supplemental Information 3 — This file contains a sample subset of EarVN1.0 that includes selected ear images from female participants (sampled from Person IDs 99–164). The images are chosen to represent variations in pose, illumination, and environmental settings, offering a comprehensive foundation for female ear recognition studies. [file peerj-cs-10-2603-s003.zip › 099.Amber/099 (12).jpg]

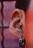

Supplement: Supplemental Information 3 — This file contains a sample subset of EarVN1.0 that includes selected ear images from female participants (sampled from Person IDs 99–164). The images are chosen to represent variations in pose, illumination, and environmental settings, offering a comprehensive foundation for female ear recognition studies. [file peerj-cs-10-2603-s003.zip › 099.Amber/099 (120).jpg]

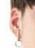

Supplement: Supplemental Information 3 — This file contains a sample subset of EarVN1.0 that includes selected ear images from female participants (sampled from Person IDs 99–164). The images are chosen to represent variations in pose, illumination, and environmental settings, offering a comprehensive foundation for female ear recognition studies. [file peerj-cs-10-2603-s003.zip › 099.Amber/099 (121).jpg]

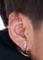

Supplement: Supplemental Information 3 — This file contains a sample subset of EarVN1.0 that includes selected ear images from female participants (sampled from Person IDs 99–164). The images are chosen to represent variations in pose, illumination, and environmental settings, offering a comprehensive foundation for female ear recognition studies. [file peerj-cs-10-2603-s003.zip › 099.Amber/099 (122).jpg]

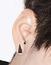

Supplement: Supplemental Information 3 — This file contains a sample subset of EarVN1.0 that includes selected ear images from female participants (sampled from Person IDs 99–164). The images are chosen to represent variations in pose, illumination, and environmental settings, offering a comprehensive foundation for female ear recognition studies. [file peerj-cs-10-2603-s003.zip › 099.Amber/099 (123).jpg]

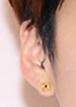

Supplement: Supplemental Information 3 — This file contains a sample subset of EarVN1.0 that includes selected ear images from female participants (sampled from Person IDs 99–164). The images are chosen to represent variations in pose, illumination, and environmental settings, offering a comprehensive foundation for female ear recognition studies. [file peerj-cs-10-2603-s003.zip › 099.Amber/099 (124).jpg]

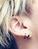

Supplement: Supplemental Information 3 — This file contains a sample subset of EarVN1.0 that includes selected ear images from female participants (sampled from Person IDs 99–164). The images are chosen to represent variations in pose, illumination, and environmental settings, offering a comprehensive foundation for female ear recognition studies. [file peerj-cs-10-2603-s003.zip › 099.Amber/099 (125).jpg]

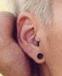

Supplement: Supplemental Information 3 — This file contains a sample subset of EarVN1.0 that includes selected ear images from female participants (sampled from Person IDs 99–164). The images are chosen to represent variations in pose, illumination, and environmental settings, offering a comprehensive foundation for female ear recognition studies. [file peerj-cs-10-2603-s003.zip › 099.Amber/099 (126).jpg]

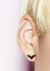

Supplement: Supplemental Information 3 — This file contains a sample subset of EarVN1.0 that includes selected ear images from female participants (sampled from Person IDs 99–164). The images are chosen to represent variations in pose, illumination, and environmental settings, offering a comprehensive foundation for female ear recognition studies. [file peerj-cs-10-2603-s003.zip › 099.Amber/099 (127).jpg]

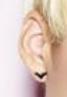

Supplement: Supplemental Information 3 — This file contains a sample subset of EarVN1.0 that includes selected ear images from female participants (sampled from Person IDs 99–164). The images are chosen to represent variations in pose, illumination, and environmental settings, offering a comprehensive foundation for female ear recognition studies. [file peerj-cs-10-2603-s003.zip › 099.Amber/099 (128).jpg]

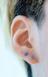

Supplement: Supplemental Information 3 — This file contains a sample subset of EarVN1.0 that includes selected ear images from female participants (sampled from Person IDs 99–164). The images are chosen to represent variations in pose, illumination, and environmental settings, offering a comprehensive foundation for female ear recognition studies. [file peerj-cs-10-2603-s003.zip › 099.Amber/099 (129).jpg]

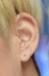

Supplement: Supplemental Information 3 — This file contains a sample subset of EarVN1.0 that includes selected ear images from female participants (sampled from Person IDs 99–164). The images are chosen to represent variations in pose, illumination, and environmental settings, offering a comprehensive foundation for female ear recognition studies. [file peerj-cs-10-2603-s003.zip › 099.Amber/099 (13).jpg]

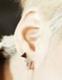

Supplement: Supplemental Information 3 — This file contains a sample subset of EarVN1.0 that includes selected ear images from female participants (sampled from Person IDs 99–164). The images are chosen to represent variations in pose, illumination, and environmental settings, offering a comprehensive foundation for female ear recognition studies. [file peerj-cs-10-2603-s003.zip › 099.Amber/099 (130).jpg]

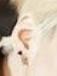

Supplement: Supplemental Information 3 — This file contains a sample subset of EarVN1.0 that includes selected ear images from female participants (sampled from Person IDs 99–164). The images are chosen to represent variations in pose, illumination, and environmental settings, offering a comprehensive foundation for female ear recognition studies. [file peerj-cs-10-2603-s003.zip › 099.Amber/099 (131).jpg]

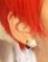

Supplement: Supplemental Information 3 — This file contains a sample subset of EarVN1.0 that includes selected ear images from female participants (sampled from Person IDs 99–164). The images are chosen to represent variations in pose, illumination, and environmental settings, offering a comprehensive foundation for female ear recognition studies. [file peerj-cs-10-2603-s003.zip › 099.Amber/099 (132).jpg]

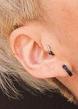

Supplement: Supplemental Information 3 — This file contains a sample subset of EarVN1.0 that includes selected ear images from female participants (sampled from Person IDs 99–164). The images are chosen to represent variations in pose, illumination, and environmental settings, offering a comprehensive foundation for female ear recognition studies. [file peerj-cs-10-2603-s003.zip › 099.Amber/099 (133).jpg]

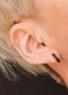

Supplement: Supplemental Information 3 — This file contains a sample subset of EarVN1.0 that includes selected ear images from female participants (sampled from Person IDs 99–164). The images are chosen to represent variations in pose, illumination, and environmental settings, offering a comprehensive foundation for female ear recognition studies. [file peerj-cs-10-2603-s003.zip › 099.Amber/099 (134).jpg]

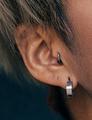

Supplement: Supplemental Information 3 — This file contains a sample subset of EarVN1.0 that includes selected ear images from female participants (sampled from Person IDs 99–164). The images are chosen to represent variations in pose, illumination, and environmental settings, offering a comprehensive foundation for female ear recognition studies. [file peerj-cs-10-2603-s003.zip › 099.Amber/099 (135).jpg]

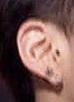

Supplement: Supplemental Information 3 — This file contains a sample subset of EarVN1.0 that includes selected ear images from female participants (sampled from Person IDs 99–164). The images are chosen to represent variations in pose, illumination, and environmental settings, offering a comprehensive foundation for female ear recognition studies. [file peerj-cs-10-2603-s003.zip › 099.Amber/099 (136).jpg]

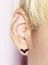

Supplement: Supplemental Information 3 — This file contains a sample subset of EarVN1.0 that includes selected ear images from female participants (sampled from Person IDs 99–164). The images are chosen to represent variations in pose, illumination, and environmental settings, offering a comprehensive foundation for female ear recognition studies. [file peerj-cs-10-2603-s003.zip › 099.Amber/099 (137).jpg]

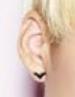

Supplement: Supplemental Information 3 — This file contains a sample subset of EarVN1.0 that includes selected ear images from female participants (sampled from Person IDs 99–164). The images are chosen to represent variations in pose, illumination, and environmental settings, offering a comprehensive foundation for female ear recognition studies. [file peerj-cs-10-2603-s003.zip › 099.Amber/099 (138).jpg]

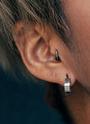

Supplement: Supplemental Information 3 — This file contains a sample subset of EarVN1.0 that includes selected ear images from female participants (sampled from Person IDs 99–164). The images are chosen to represent variations in pose, illumination, and environmental settings, offering a comprehensive foundation for female ear recognition studies. [file peerj-cs-10-2603-s003.zip › 099.Amber/099 (139).jpg]

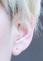

Supplement: Supplemental Information 3 — This file contains a sample subset of EarVN1.0 that includes selected ear images from female participants (sampled from Person IDs 99–164). The images are chosen to represent variations in pose, illumination, and environmental settings, offering a comprehensive foundation for female ear recognition studies. [file peerj-cs-10-2603-s003.zip › 099.Amber/099 (14).jpg]

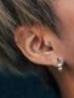

Supplement: Supplemental Information 3 — This file contains a sample subset of EarVN1.0 that includes selected ear images from female participants (sampled from Person IDs 99–164). The images are chosen to represent variations in pose, illumination, and environmental settings, offering a comprehensive foundation for female ear recognition studies. [file peerj-cs-10-2603-s003.zip › 099.Amber/099 (140).jpg]

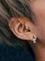

Supplement: Supplemental Information 3 — This file contains a sample subset of EarVN1.0 that includes selected ear images from female participants (sampled from Person IDs 99–164). The images are chosen to represent variations in pose, illumination, and environmental settings, offering a comprehensive foundation for female ear recognition studies. [file peerj-cs-10-2603-s003.zip › 099.Amber/099 (141).jpg]

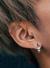

Supplement: Supplemental Information 3 — This file contains a sample subset of EarVN1.0 that includes selected ear images from female participants (sampled from Person IDs 99–164). The images are chosen to represent variations in pose, illumination, and environmental settings, offering a comprehensive foundation for female ear recognition studies. [file peerj-cs-10-2603-s003.zip › 099.Amber/099 (142).jpg]

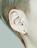

Supplement: Supplemental Information 3 — This file contains a sample subset of EarVN1.0 that includes selected ear images from female participants (sampled from Person IDs 99–164). The images are chosen to represent variations in pose, illumination, and environmental settings, offering a comprehensive foundation for female ear recognition studies. [file peerj-cs-10-2603-s003.zip › 099.Amber/099 (143).jpg]

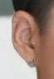

Supplement: Supplemental Information 3 — This file contains a sample subset of EarVN1.0 that includes selected ear images from female participants (sampled from Person IDs 99–164). The images are chosen to represent variations in pose, illumination, and environmental settings, offering a comprehensive foundation for female ear recognition studies. [file peerj-cs-10-2603-s003.zip › 099.Amber/099 (144).jpg]

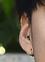

Supplement: Supplemental Information 3 — This file contains a sample subset of EarVN1.0 that includes selected ear images from female participants (sampled from Person IDs 99–164). The images are chosen to represent variations in pose, illumination, and environmental settings, offering a comprehensive foundation for female ear recognition studies. [file peerj-cs-10-2603-s003.zip › 099.Amber/099 (145).jpg]

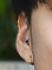

Supplement: Supplemental Information 3 — This file contains a sample subset of EarVN1.0 that includes selected ear images from female participants (sampled from Person IDs 99–164). The images are chosen to represent variations in pose, illumination, and environmental settings, offering a comprehensive foundation for female ear recognition studies. [file peerj-cs-10-2603-s003.zip › 099.Amber/099 (146).jpg]

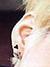

Supplement: Supplemental Information 3 — This file contains a sample subset of EarVN1.0 that includes selected ear images from female participants (sampled from Person IDs 99–164). The images are chosen to represent variations in pose, illumination, and environmental settings, offering a comprehensive foundation for female ear recognition studies. [file peerj-cs-10-2603-s003.zip › 099.Amber/099 (147).jpg]

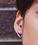

Supplement: Supplemental Information 3 — This file contains a sample subset of EarVN1.0 that includes selected ear images from female participants (sampled from Person IDs 99–164). The images are chosen to represent variations in pose, illumination, and environmental settings, offering a comprehensive foundation for female ear recognition studies. [file peerj-cs-10-2603-s003.zip › 099.Amber/099 (148).jpg]

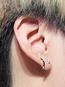

Supplement: Supplemental Information 3 — This file contains a sample subset of EarVN1.0 that includes selected ear images from female participants (sampled from Person IDs 99–164). The images are chosen to represent variations in pose, illumination, and environmental settings, offering a comprehensive foundation for female ear recognition studies. [file peerj-cs-10-2603-s003.zip › 099.Amber/099 (149).jpg]

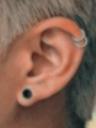

Supplement: Supplemental Information 3 — This file contains a sample subset of EarVN1.0 that includes selected ear images from female participants (sampled from Person IDs 99–164). The images are chosen to represent variations in pose, illumination, and environmental settings, offering a comprehensive foundation for female ear recognition studies. [file peerj-cs-10-2603-s003.zip › 099.Amber/099 (15).jpg]

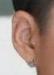

Supplement: Supplemental Information 3 — This file contains a sample subset of EarVN1.0 that includes selected ear images from female participants (sampled from Person IDs 99–164). The images are chosen to represent variations in pose, illumination, and environmental settings, offering a comprehensive foundation for female ear recognition studies. [file peerj-cs-10-2603-s003.zip › 099.Amber/099 (150).jpg]

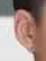

Supplement: Supplemental Information 3 — This file contains a sample subset of EarVN1.0 that includes selected ear images from female participants (sampled from Person IDs 99–164). The images are chosen to represent variations in pose, illumination, and environmental settings, offering a comprehensive foundation for female ear recognition studies. [file peerj-cs-10-2603-s003.zip › 099.Amber/099 (151).jpg]

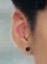

Supplement: Supplemental Information 3 — This file contains a sample subset of EarVN1.0 that includes selected ear images from female participants (sampled from Person IDs 99–164). The images are chosen to represent variations in pose, illumination, and environmental settings, offering a comprehensive foundation for female ear recognition studies. [file peerj-cs-10-2603-s003.zip › 099.Amber/099 (152).jpg]

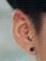

Supplement: Supplemental Information 3 — This file contains a sample subset of EarVN1.0 that includes selected ear images from female participants (sampled from Person IDs 99–164). The images are chosen to represent variations in pose, illumination, and environmental settings, offering a comprehensive foundation for female ear recognition studies. [file peerj-cs-10-2603-s003.zip › 099.Amber/099 (153).jpg]

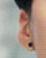

Supplement: Supplemental Information 3 — This file contains a sample subset of EarVN1.0 that includes selected ear images from female participants (sampled from Person IDs 99–164). The images are chosen to represent variations in pose, illumination, and environmental settings, offering a comprehensive foundation for female ear recognition studies. [file peerj-cs-10-2603-s003.zip › 099.Amber/099 (154).jpg]

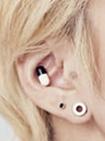

Supplement: Supplemental Information 3 — This file contains a sample subset of EarVN1.0 that includes selected ear images from female participants (sampled from Person IDs 99–164). The images are chosen to represent variations in pose, illumination, and environmental settings, offering a comprehensive foundation for female ear recognition studies. [file peerj-cs-10-2603-s003.zip › 099.Amber/099 (155).jpg]

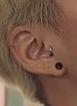

Supplement: Supplemental Information 3 — This file contains a sample subset of EarVN1.0 that includes selected ear images from female participants (sampled from Person IDs 99–164). The images are chosen to represent variations in pose, illumination, and environmental settings, offering a comprehensive foundation for female ear recognition studies. [file peerj-cs-10-2603-s003.zip › 099.Amber/099 (156).jpg]

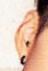

Supplement: Supplemental Information 3 — This file contains a sample subset of EarVN1.0 that includes selected ear images from female participants (sampled from Person IDs 99–164). The images are chosen to represent variations in pose, illumination, and environmental settings, offering a comprehensive foundation for female ear recognition studies. [file peerj-cs-10-2603-s003.zip › 099.Amber/099 (157).jpg]

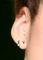

Supplement: Supplemental Information 3 — This file contains a sample subset of EarVN1.0 that includes selected ear images from female participants (sampled from Person IDs 99–164). The images are chosen to represent variations in pose, illumination, and environmental settings, offering a comprehensive foundation for female ear recognition studies. [file peerj-cs-10-2603-s003.zip › 099.Amber/099 (158).jpg]

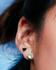

Supplement: Supplemental Information 3 — This file contains a sample subset of EarVN1.0 that includes selected ear images from female participants (sampled from Person IDs 99–164). The images are chosen to represent variations in pose, illumination, and environmental settings, offering a comprehensive foundation for female ear recognition studies. [file peerj-cs-10-2603-s003.zip › 099.Amber/099 (159).jpg]

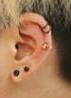

Supplement: Supplemental Information 3 — This file contains a sample subset of EarVN1.0 that includes selected ear images from female participants (sampled from Person IDs 99–164). The images are chosen to represent variations in pose, illumination, and environmental settings, offering a comprehensive foundation for female ear recognition studies. [file peerj-cs-10-2603-s003.zip › 099.Amber/099 (16).jpg]

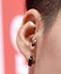

Supplement: Supplemental Information 3 — This file contains a sample subset of EarVN1.0 that includes selected ear images from female participants (sampled from Person IDs 99–164). The images are chosen to represent variations in pose, illumination, and environmental settings, offering a comprehensive foundation for female ear recognition studies. [file peerj-cs-10-2603-s003.zip › 099.Amber/099 (160).jpg]

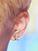

Supplement: Supplemental Information 3 — This file contains a sample subset of EarVN1.0 that includes selected ear images from female participants (sampled from Person IDs 99–164). The images are chosen to represent variations in pose, illumination, and environmental settings, offering a comprehensive foundation for female ear recognition studies. [file peerj-cs-10-2603-s003.zip › 099.Amber/099 (17).jpg]

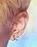

Supplement: Supplemental Information 3 — This file contains a sample subset of EarVN1.0 that includes selected ear images from female participants (sampled from Person IDs 99–164). The images are chosen to represent variations in pose, illumination, and environmental settings, offering a comprehensive foundation for female ear recognition studies. [file peerj-cs-10-2603-s003.zip › 099.Amber/099 (18).jpg]

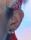

Supplement: Supplemental Information 3 — This file contains a sample subset of EarVN1.0 that includes selected ear images from female participants (sampled from Person IDs 99–164). The images are chosen to represent variations in pose, illumination, and environmental settings, offering a comprehensive foundation for female ear recognition studies. [file peerj-cs-10-2603-s003.zip › 099.Amber/099 (19).jpg]

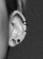

Supplement: Supplemental Information 3 — This file contains a sample subset of EarVN1.0 that includes selected ear images from female participants (sampled from Person IDs 99–164). The images are chosen to represent variations in pose, illumination, and environmental settings, offering a comprehensive foundation for female ear recognition studies. [file peerj-cs-10-2603-s003.zip › 099.Amber/099 (2).jpg]

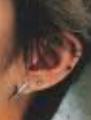

Supplement: Supplemental Information 3 — This file contains a sample subset of EarVN1.0 that includes selected ear images from female participants (sampled from Person IDs 99–164). The images are chosen to represent variations in pose, illumination, and environmental settings, offering a comprehensive foundation for female ear recognition studies. [file peerj-cs-10-2603-s003.zip › 099.Amber/099 (20).jpg]

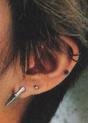

Supplement: Supplemental Information 3 — This file contains a sample subset of EarVN1.0 that includes selected ear images from female participants (sampled from Person IDs 99–164). The images are chosen to represent variations in pose, illumination, and environmental settings, offering a comprehensive foundation for female ear recognition studies. [file peerj-cs-10-2603-s003.zip › 099.Amber/099 (21).jpg]

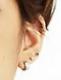

Supplement: Supplemental Information 3 — This file contains a sample subset of EarVN1.0 that includes selected ear images from female participants (sampled from Person IDs 99–164). The images are chosen to represent variations in pose, illumination, and environmental settings, offering a comprehensive foundation for female ear recognition studies. [file peerj-cs-10-2603-s003.zip › 099.Amber/099 (22).jpg]

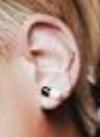

Supplement: Supplemental Information 3 — This file contains a sample subset of EarVN1.0 that includes selected ear images from female participants (sampled from Person IDs 99–164). The images are chosen to represent variations in pose, illumination, and environmental settings, offering a comprehensive foundation for female ear recognition studies. [file peerj-cs-10-2603-s003.zip › 099.Amber/099 (23).jpg]

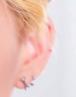

Supplement: Supplemental Information 3 — This file contains a sample subset of EarVN1.0 that includes selected ear images from female participants (sampled from Person IDs 99–164). The images are chosen to represent variations in pose, illumination, and environmental settings, offering a comprehensive foundation for female ear recognition studies. [file peerj-cs-10-2603-s003.zip › 099.Amber/099 (24).jpg]

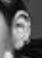

Supplement: Supplemental Information 3 — This file contains a sample subset of EarVN1.0 that includes selected ear images from female participants (sampled from Person IDs 99–164). The images are chosen to represent variations in pose, illumination, and environmental settings, offering a comprehensive foundation for female ear recognition studies. [file peerj-cs-10-2603-s003.zip › 099.Amber/099 (25).jpg]

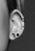

Supplement: Supplemental Information 3 — This file contains a sample subset of EarVN1.0 that includes selected ear images from female participants (sampled from Person IDs 99–164). The images are chosen to represent variations in pose, illumination, and environmental settings, offering a comprehensive foundation for female ear recognition studies. [file peerj-cs-10-2603-s003.zip › 099.Amber/099 (26).jpg]

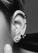

Supplement: Supplemental Information 3 — This file contains a sample subset of EarVN1.0 that includes selected ear images from female participants (sampled from Person IDs 99–164). The images are chosen to represent variations in pose, illumination, and environmental settings, offering a comprehensive foundation for female ear recognition studies. [file peerj-cs-10-2603-s003.zip › 099.Amber/099 (27).jpg]

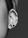

Supplement: Supplemental Information 3 — This file contains a sample subset of EarVN1.0 that includes selected ear images from female participants (sampled from Person IDs 99–164). The images are chosen to represent variations in pose, illumination, and environmental settings, offering a comprehensive foundation for female ear recognition studies. [file peerj-cs-10-2603-s003.zip › 099.Amber/099 (28).jpg]

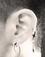

Supplement: Supplemental Information 3 — This file contains a sample subset of EarVN1.0 that includes selected ear images from female participants (sampled from Person IDs 99–164). The images are chosen to represent variations in pose, illumination, and environmental settings, offering a comprehensive foundation for female ear recognition studies. [file peerj-cs-10-2603-s003.zip › 099.Amber/099 (29).jpg]

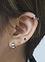

Supplement: Supplemental Information 3 — This file contains a sample subset of EarVN1.0 that includes selected ear images from female participants (sampled from Person IDs 99–164). The images are chosen to represent variations in pose, illumination, and environmental settings, offering a comprehensive foundation for female ear recognition studies. [file peerj-cs-10-2603-s003.zip › 099.Amber/099 (3).jpg]

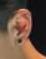

Supplement: Supplemental Information 3 — This file contains a sample subset of EarVN1.0 that includes selected ear images from female participants (sampled from Person IDs 99–164). The images are chosen to represent variations in pose, illumination, and environmental settings, offering a comprehensive foundation for female ear recognition studies. [file peerj-cs-10-2603-s003.zip › 099.Amber/099 (30).jpg]

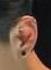

Supplement: Supplemental Information 3 — This file contains a sample subset of EarVN1.0 that includes selected ear images from female participants (sampled from Person IDs 99–164). The images are chosen to represent variations in pose, illumination, and environmental settings, offering a comprehensive foundation for female ear recognition studies. [file peerj-cs-10-2603-s003.zip › 099.Amber/099 (31).jpg]

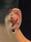

Supplement: Supplemental Information 3 — This file contains a sample subset of EarVN1.0 that includes selected ear images from female participants (sampled from Person IDs 99–164). The images are chosen to represent variations in pose, illumination, and environmental settings, offering a comprehensive foundation for female ear recognition studies. [file peerj-cs-10-2603-s003.zip › 099.Amber/099 (32).jpg]

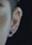

Supplement: Supplemental Information 3 — This file contains a sample subset of EarVN1.0 that includes selected ear images from female participants (sampled from Person IDs 99–164). The images are chosen to represent variations in pose, illumination, and environmental settings, offering a comprehensive foundation for female ear recognition studies. [file peerj-cs-10-2603-s003.zip › 099.Amber/099 (33).jpg]

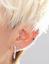

Supplement: Supplemental Information 3 — This file contains a sample subset of EarVN1.0 that includes selected ear images from female participants (sampled from Person IDs 99–164). The images are chosen to represent variations in pose, illumination, and environmental settings, offering a comprehensive foundation for female ear recognition studies. [file peerj-cs-10-2603-s003.zip › 099.Amber/099 (34).jpg]

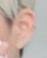

Supplement: Supplemental Information 3 — This file contains a sample subset of EarVN1.0 that includes selected ear images from female participants (sampled from Person IDs 99–164). The images are chosen to represent variations in pose, illumination, and environmental settings, offering a comprehensive foundation for female ear recognition studies. [file peerj-cs-10-2603-s003.zip › 099.Amber/099 (35).jpg]

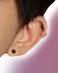

Supplement: Supplemental Information 3 — This file contains a sample subset of EarVN1.0 that includes selected ear images from female participants (sampled from Person IDs 99–164). The images are chosen to represent variations in pose, illumination, and environmental settings, offering a comprehensive foundation for female ear recognition studies. [file peerj-cs-10-2603-s003.zip › 099.Amber/099 (36).jpg]

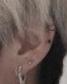

Supplement: Supplemental Information 3 — This file contains a sample subset of EarVN1.0 that includes selected ear images from female participants (sampled from Person IDs 99–164). The images are chosen to represent variations in pose, illumination, and environmental settings, offering a comprehensive foundation for female ear recognition studies. [file peerj-cs-10-2603-s003.zip › 099.Amber/099 (37).jpg]

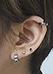

Supplement: Supplemental Information 3 — This file contains a sample subset of EarVN1.0 that includes selected ear images from female participants (sampled from Person IDs 99–164). The images are chosen to represent variations in pose, illumination, and environmental settings, offering a comprehensive foundation for female ear recognition studies. [file peerj-cs-10-2603-s003.zip › 099.Amber/099 (38).jpg]

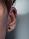

Supplement: Supplemental Information 3 — This file contains a sample subset of EarVN1.0 that includes selected ear images from female participants (sampled from Person IDs 99–164). The images are chosen to represent variations in pose, illumination, and environmental settings, offering a comprehensive foundation for female ear recognition studies. [file peerj-cs-10-2603-s003.zip › 099.Amber/099 (39).jpg]

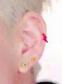

Supplement: Supplemental Information 3 — This file contains a sample subset of EarVN1.0 that includes selected ear images from female participants (sampled from Person IDs 99–164). The images are chosen to represent variations in pose, illumination, and environmental settings, offering a comprehensive foundation for female ear recognition studies. [file peerj-cs-10-2603-s003.zip › 099.Amber/099 (4).jpg]

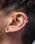

Supplement: Supplemental Information 3 — This file contains a sample subset of EarVN1.0 that includes selected ear images from female participants (sampled from Person IDs 99–164). The images are chosen to represent variations in pose, illumination, and environmental settings, offering a comprehensive foundation for female ear recognition studies. [file peerj-cs-10-2603-s003.zip › 099.Amber/099 (40).jpg]

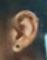

Supplement: Supplemental Information 3 — This file contains a sample subset of EarVN1.0 that includes selected ear images from female participants (sampled from Person IDs 99–164). The images are chosen to represent variations in pose, illumination, and environmental settings, offering a comprehensive foundation for female ear recognition studies. [file peerj-cs-10-2603-s003.zip › 099.Amber/099 (41).jpg]

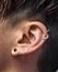

Supplement: Supplemental Information 3 — This file contains a sample subset of EarVN1.0 that includes selected ear images from female participants (sampled from Person IDs 99–164). The images are chosen to represent variations in pose, illumination, and environmental settings, offering a comprehensive foundation for female ear recognition studies. [file peerj-cs-10-2603-s003.zip › 099.Amber/099 (42).jpg]

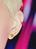

Supplement: Supplemental Information 3 — This file contains a sample subset of EarVN1.0 that includes selected ear images from female participants (sampled from Person IDs 99–164). The images are chosen to represent variations in pose, illumination, and environmental settings, offering a comprehensive foundation for female ear recognition studies. [file peerj-cs-10-2603-s003.zip › 099.Amber/099 (43).jpg]

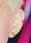

Supplement: Supplemental Information 3 — This file contains a sample subset of EarVN1.0 that includes selected ear images from female participants (sampled from Person IDs 99–164). The images are chosen to represent variations in pose, illumination, and environmental settings, offering a comprehensive foundation for female ear recognition studies. [file peerj-cs-10-2603-s003.zip › 099.Amber/099 (44).jpg]
