# Supplementary material for: DANNET: deep attention neural network for efficient ear identification in biometrics
Source: PeerJ Comput Sci. 2024 Dec 18;10:e2603. doi: 10.7717/peerj-cs.2603 (PMC11784740; doi:10.7717/peerj-cs.2603)
Supplement: Supplemental Information 4 [file peerj-cs-10-2603-s004.pdf]

```python
import time
import tensorflow as tf

# Start time for training
start_train_time = time.time()

# Training code here (e.g., model.fit)
model.fit(train_data, train_labels, epochs=50,
validation_data=(val_data, val_labels))

# End time for training
end_train_time = time.time()

# Calculate training time
training_time = end_train_time - start_train_time

# Start time for testing
start_test_time = time.time()

# Testing code here (e.g., model.evaluate)
test_loss, test_accuracy = model.evaluate(test_data, test_labels)

# End time for testing
end_test_time = time.time()

# Calculate testing time
testing_time = end_test_time - start_test_time

# Print the times
print(f"Training Time: {training_time:.2f} seconds")
print(f"Testing Time: {testing_time:.2f} seconds")

# If multiple runs, compute average time

total_testing_time = testing_time_1 + testing_time_2 + testing_time_3
average_testing_time = total_testing_time / 3

print(f"Average Testing Time: {average_testing_time:.2f} seconds")
```
